# Deciphering Tryptophan Oxygenation: Key Modulators of 2-Oxindole Formation in MarE

**DOI:** 10.1002/anie.202510848

**Published:** 2025-07-20

**Authors:** Romie C. Nguyen, Inchul Shin, Aimin Liu

**Affiliations:** Department of Chemistry, The University of Texas at San Antonio, Texas, U.S.A.

**Keywords:** Dioxygenase, Enzyme Mechanism, Monooxygenase, Natural product biosynthesis, Oxindole

## Abstract

MarE, a heme-dependent aromatic oxygenase with a histidyl axial ligation, catalyzes the monooxygenation of *β*-methyl-l-tryptophan to form a 2-oxindole scaffold central to maremycin biosynthesis. Although structurally similar to tryptophan 2,3-dioxygenase (TDO), which initiates l-tryptophan catabolism via dioxygenation, MarE exhibits distinct reactivity modulated by ascorbate. While ascorbate has no effect on TDO, it promotes selective monooxygenation by MarE. In its absence, MarE favors dioxygenation and formation of pyrroloindoline products, revealing latent catalytic versatility. Active-site loop sequences differ between the two enzymes, SLGGR in MarE versus GTGGS in TDO, prompting loop-swapping experiments to probe structure-function relationships. Substituting GTGGS in TDO to MarE-like sequences (GTGGA or SLGGS) shifted reactivity toward monooxygenation and formation of C3-hydroxylated, non-oxindole products that underwent further cyclization into tricyclic structures. Conversely, replacing SLGGR in MarE with GTGGS resulted in enhanced C2,C3-dioxygenation nearly 4-fold. These results underscore the active-site loop as a key determinant of oxidation outcome, alongside the modulatory role of ascorbate. By revealing the true catalytic identity of MarE and delineating the roles of small-molecule effectors and loop architecture, this study advances mechanistic understanding and predictive capabilities within the oxygenase superfamily.

## Graphical Abstract

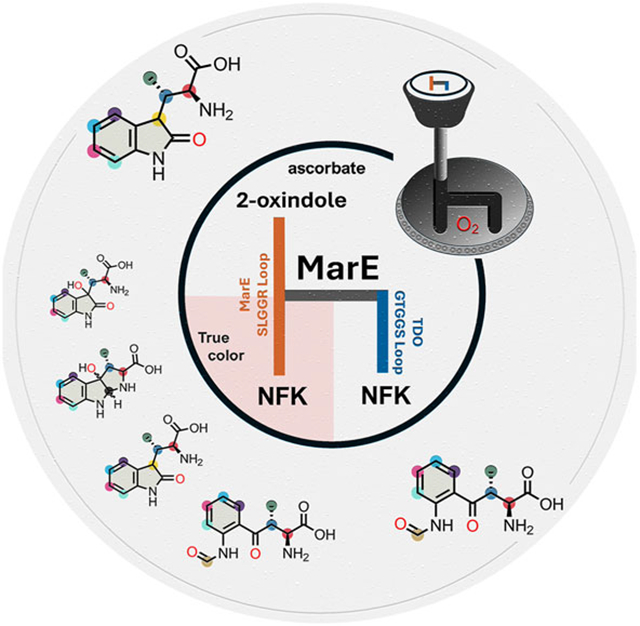

This study reveals that MarE, a heme-dependent aromatic oxygenase, favors dioxygenation of *β*-methyl-l-tryptophan in the absence of ascorbate and demonstrates how structural and redox tuning shifts its reactivity toward selective monooxygenation, yielding a 2-oxindole scaffold. These findings offer new insights into enzyme-controlled indole oxidation in tryptophan-derived natural products.

The aromatic amino acid tryptophan plays a wide variety of roles in nature and serves as an important precursor for synthesizing proteins, neurotransmitters, their regulators, redox carriers, and natural products. Tryptophan dioxygenases, such as indoleamine 2,3-dioxygenase (IDO) and tryptophan 2,3-dioxygenase (TDO), catalyze the heme-dependent insertion of molecular oxygen into the indole ring of their substrate, l-tryptophan (l-Trp, **2**) producing *N*-formylkynurenine (NFK, **2d**), without the need for additional electrons and protons (Scheme 1a).^[1–2]^

Recent bioinformatic analyses and structural alignments have revealed that these tryptophan dioxygenase enzymes are part of a larger protein group known as the histidyl-ligated heme-dependent aromatic oxygenase (HDAO) superfamily.^[3]^ This structure-based, functionally related superfamily includes thousands of members with subgroups that oxidize tryptophan or tyrosine and their metabolites. Some, like IDO/TDO, initiate tryptophan catabolism and serve as immune checkpoints, exploited by cancers for immune evasion.^[4–5]^ Others, such as MarE, SfmD, and TyrH, are critical in building the core structure of bioactive natural products from amino acids in secondary metabolism.^[6–10]^ Thus, the functions of HDAO members exhibit substantial implications across multiple scientific domains.

MarE is a recently identified enzyme that, in the presence of ascorbate, catalyzes the monooxygenation of (2*S*,3*S*)-*β*-methyl-l-tryptophan (*β*-Me-l-Trp, **1**) to produce a unique 2-oxindole (**1a**) product (Scheme 1b).^[6]^ The crystal structure of this enzyme firmly assigns MarE as a member of the HDAO superfamily.^[11]^ The 2-oxindole moiety serves as a key scaffold for the synthesis of a variety of bioactive compounds,^[6,12]^ such as maremycins (Scheme 1c), which exhibit antimicrobial activity, and spirooxindoles, which serve as a promising scaffold for anticancer agents (Scheme 1d).^[6,13–14]^

In the enzyme-substrate complex crystal structure, *β*-Me-l-Trp binds to the distal heme pocket of MarE analogous to how l-Trp binds in the active site of IDO/TDO.^[11]^ Both enzymes share similar protein structure and substrate interaction modes. However, the structural advancements do not address the intriguing reaction outcomes of these enzymes. Specifically, it remains unclear why MarE functions as a monooxygenase enzyme that catalyzes a single C2 oxygenation,^[6,11]^ whereas IDO/TDO produces a C2,C3-dioxygenated product (**2d**) through a stepwise O-atom transfer reaction process,^[15–18]^ which is a common conundrum in these enzymes and synthetic model complexes.^[19–22]^

To tackle this enigma, we conducted a detailed analysis of the MarE-catalyzed reaction to elucidate the governing factors for its preference for 2-oxindole formation. In the O_2_-dependent monooxygenation, *β*-Me-l-Trp provides 2e^−^ to O_2_. The overall reaction requires an additional 2e^−^ and 2H^+^ to reduce O_2_, suggesting an external electron donor, i.e., a cosubstrate, is missing from the reaction of MarE. We then conducted the reaction with and without an additional reducing agent to determine its role in the process. This approach aims to provide a comprehensive understanding of the reaction components.

We observed five reaction products using a full-spectrum HPLC detector when examining the wild-type ferrous heme-containing MarE reaction profile with **1** in oxygen-saturated buffer and without any cosubstrate (Figure 1a). The product **1a** was a monooxygenated result with all the characteristics identical to the previously reported 2-oxindole product. Surprisingly, the primary reaction product (**1b**) and a minor product **1b**_**1**_ were dioxygenated products, each exhibiting a 32-Da increase from **1** (Figure 1b).

The chemical structures of those newly identified MarE reaction products were determined by NMR (Figure 2). Product **1b** is a mixture of stereoisomers of the NFK-like pyrrole ring-opened dioxygenated products at 1:1.3 ratio (Figure S1 and Table S2). Product **1b**_**1**_ is a dioxygenated product containing a 2-oxindole ketone moiety and an additional hydroxylation at C3 (Figure S2 and Table S3). Products **1a**_**1**_ and **1a**_**2**_ are diastereomers containing a C3-monooxygenated tricyclic pyrroloindoline structure similar to the previously identified TDO C3-monooxygenated furoindoline product, utilizing a substrate analog of **2** that substitutes the *α*-amino nitrogen with oxygen (Figure S3 and Table S4).^[18]^ The structure of **1a**_**1**_ appears to be an isomer of **1a**_**2**_, with the chemical shift of its C2 proton appearing slightly more upfield compared to **1a**_**2**_ (Figure S4 and Table S5). Together, these results reveal the true colors of MarE’s catalytic versatility, producing monooxygenated, dioxygenated, and pyrroloindoline products.

Based on these results, an additional factor outside the enzyme must be present to drive the reaction toward monooxygenation. We revisited common electron donors, and our results indicated that the monooxygenated 2-oxindole product **1a** was predominantly produced from **1** in the presence of ascorbate (Figure 3, inset), echoing the previous study.^[6]^ However, unlike the previous report, we detected a minor dioxygenated product **1b** exhibiting the characteristic UV–vis spectral feature of NFK at 322 nm and a 32 Da mass increase (Figure S5). A second minor monooxygenated product **1c** eluted the earliest, sharing similar UV–vis spectral features and *m/z* of 235 (Figure S6) with **1a** in a much-reduced quantity (Figure S5). These results suggest that an electron donor such as ascorbate is a critical factor in driving MarE toward monooxygenation but remains insufficient to function as the sole factor responsible for single oxygen insertion, specifically at the C2 position. The product distribution shift observed with ascorbate raises questions about its physiological relevance. Notably, *Streptomyces sp*. B9173 (also known as *S. rishiriensis*), the native producer of MarE, encodes a homolog of rat l-gulonolactone oxidase—the terminal enzyme in the biosynthetic pathway converting l-gulono-*γ*-lactone to ascorbate. This implies that the organism may endogenously generate ascorbate or a similar reductant. Importantly, in the absence of ascorbate, MarE yields a broader set of oxygenated products, many lacking the 2-oxindole moiety observed under reducing conditions. Thus, ascorbate not only modulates product selectivity but may also reflect a native cofactor environment relevant to MarE’s physiological function.

Next, we explored additional factors influencing reaction outcomes. Structural comparison between MarE with IDO and TDO revealed that the SLGGR loop of MarE (residues 231 to 235) corresponds to the conserved GTGGS sequences in IDO/TDO in the catalytic active site (Figures 4a and S7). The GTGGS (residues 341 to 345 in human TDO numbering) is part of the large JK-loop,^[1]^ which bridges the substrate amino group to the heme propionate positioned above the heme plane (hereafter, the up-propionate) (Figure 4b). Notably, the GTGGS loop forms an H-bond with the *α*-amino nitrogen of l-Trp (Figure 4b),^[23–25]^ suggesting a catalytic role for this loop in IDO and TDO, though its precise impact on the reaction remains unclear.

We then experimentally examined how loop differences influence catalysis in MarE and human TDO. A single-point mutation in the right end of the TDO loop (GTGGS-to-GTGGA) enhanced monooxygenation when using indole-3-propionic acid (IPA, **3**), a mechanistic probe lacking the *α*-amino group of l-Trp (Figure S8). Compared to wild-type TDO, this variant produced ~1.4-fold more monooxygenated 2-oxindole product **3b** (Figure S8 and Scheme 2). However, when tested with l-Trp (**2**), the single-point mutation had no discernible impact, yielding a product distribution nearly identical to wild-type TDO (Figure S9).

We then tested Thr342, which is H-bonded to the substrate. A double mutation (GTGGS-to-SLGGS) at the loop’s front end reduced dioxygenation efficiency toward l-Trp. The reaction of this variant primarily yielded **2d**, the expected NFK product (~95%), but also generated a minor (~5%) monooxygenated product **2e** distinct from the 2-oxindole observed in MarE (Figure 5 and Figure S10). The UV–vis spectrum of this minor product **2e** aligns well with **1a**_**1**_, **1a**_**2**_, and **4**, consistent with previous TDO reactions using an *N*-to-*O* substituted l-Trp analog (Figure 5 inset), whose chemical structure has been fully elucidated by HRMS and NMR.^[18]^ Thus, **2e** underwent hydroxylation at C3, triggering spontaneous cyclization into a tricyclic structure as previously found for **4**. Using the native substrate of MarE (**1**), this TDO variant produced three products: a monooxygenated species **1e**, though not a 2-oxindole, and two NFK-like dioxygenated products, **1d** and **1d**′ (Figure S11 and Scheme 2).

Challenging MarE with the non-native substrate l-Trp (**2**) resulted in cross-reactivity and complex product distribution (Figure S12A). Two monooxygenated products, **2a** and **2a′** exhibited UV–vis spectral features similar to the 2-oxindole-containing product **1a** (Figure 2 and S12B), and their identical *m/z* values (221) confirmed them as 2-oxindole derivatives of **2** (Figure S12C and Scheme 2). However, the SLGGR-to-GTGGR MarE variant showed no significant change in the distribution of mono- and dioxygenated products compared to wild-type MarE (Figures S12A and S5A), indicating that the SLGGR loop composition in MarE has a less pronounced impact than its counterpart in TDO. Structural analysis further supported this, as the *α*-amino group of **1** does not interact with this loop (Figure 4a).

The reciprocal SLGGR-to-GTGGS loop swap triple mutation in MarE, which fully mimics the TDO loop, increased C3 oxygenation in the presence of ascorbate, lead to a 1.6-fold increase in the C2,C3-dioxygenated product **1b** (Figure 3). The reaction of this mutant in the absence of ascorbate resulted in a near 4-fold increase of dioxygenated product **1b**. This substantial shift underscores the critical role of the loop region in promoting dioxygenation chemistry and highlights that the observed effect is not solely attributable to ascorbate but rather to the structural mimicry of the TDO active site loop. This loop likely plays a direct role in controlling substrate positioning and O_2_ access, thereby influencing whether a mono- or dioxygenation event occurs. When this loop is mutated to mimic the GTGGS motif found in TDO, the production of the NFK-like dioxygenation product markedly increases—even in the absence of ascorbate—supporting the idea that loop dynamics and structural geometry are central to oxygenation outcomes. These loop studies illustrate that this region plays a crucial role in directing mono- versus dioxygenation chemistry, with its influence being substrate-dependent (i.e., influenced by the presence or absence of the *α*-amino group), enzyme-context-dependent due to variations in loop composition, and ascorbate dependent.

To further evaluate structural contributions to oxygenation outcomes and enhance scientific rigor, we examined differences in other regions including near the heme propionate below the plane (the down-propionate) (Figure S13). In TDO, Arg159 and Tyr350 directly interact with the down-propionate, while Arg325, positioned 3.5 Å away, may contribute indirectly to the heme environment, either through electrostatic interactions with the heme propionate or via bridging water molecules. Such distal interactions are commonly observed in heme enzymes and are known to influence heme orientation, redox properties, and reactivity. In contrast, MarE’s down-propionate interacts solely with Arg243.

R159A, Y350F, and R325A mutants in TDO were generated. When challenged with l-Trp (**2**), these mutations had minimal impact, as all variants produced similar amounts of NFK (**2d**) as the wild-type enzyme (Figure S14). However, with IPA (**3**), these TDO mutants exhibited increased monooxygenation. Compared to wild-type TDO, Y350F, R159A, and R325A produced approximately 3.8-, 3.7-, and 1.5-fold more of the monooxygenated product **3b**, respectively (Figure S15). In contrast, the TDO loop variants, except the single-point mutation, did not enhance the **3b** formation (Figure S8). These indicate that the down-propionate region in TDO plays a role in substrate selectivity and product outcome, particularly when the substrate lacks an *α*-amino group, as previously expected for another HDAO enzyme TyrH.^[26]^ All generated TDO variants exhibited reduced production of dioxygenated product **3c** relative to wild-type enzyme (Figures S8, S15, and S17). Similarly, MarE loop variants produced more **3b** and less **3c** than wild-type MarE (Figures S15–S17), suggesting that the TDO-mimicking loop in MarE has little influence when the substrate lacks an *α*-amino group. These variant studies indicate that modifications in the down-propionate region led to shifts in reactivity with IPA (**3**) versus *β*-Me-l-Trp (**1**), suggesting that the region may indirectly affect substrate orientation or accessibility of reactive oxygen species, thereby contributing to substrate discrimination. While not a part of the substrate binding pocket per se, this region appears to act as a functional gatekeeper, tuning the enzyme’s reactivity landscape in a substrate-dependent manner.

This work reveals a more nuanced view of MarE’s reactivity, showing that it is not strictly a monooxygenase—as previously assumed—but functions primarily as a dioxygenase. Ascorbate emerges as the primary determinant of monooxygenation in this experimental system. The shift in product profile in the presence of ascorbate suggests that redox conditions can modulate the enzyme’s catalytic pathway, but only within the structural context of MarE. This modulation likely depends on features such as the flexible SLGGR loop adjacent to the active site. Moreover, the identification of a novel three-ring monooxygenated product connects MarE’s activity to natural product biosynthesis in *Streptomyces* sp. B9173. Given the precedent for similar scaffolds in other *Streptomyces*-derived metabolites, this enzyme may serve as a biosynthetic entry point to a broader array of structurally diverse compounds.

Our data supports a mechanistic model in which MarE’s oxygenation profile is dictated by a combination of loop-dependent substrate control and ascorbate-modulated redox flexibility—features not shared by IDO/TDO and other known HDAO enzymes involved in metabolizing tryptophan and its derivatives. Ascorbate emerges as the primary determinant of monooxygenation in this experimental system, while active-site features—such as the SLGGR loop in MarE and the GTGGS loop in IDO/TDO—fine-tune oxidation site selectivity. In the presence of ascorbate, MarE predominantly enforces preferential C2 monooxygenation of *β*-Me-l-Trp (**1**), whereas IDO/TDO are guided by their distinct loop architecture and facilitates sequential C3 and C2 dioxygenation of l-Trp (**2**). The structure-function relationship described here highlights broader implications for heme-enzyme-mediated aromatic amino acid oxidations in metabolism and natural product biosynthesis.

## Supplementary Material

Supporting Information

Materials and Methods, (Tables S1–S5), (Figures S1–S17), and associated references.

Additional supporting information can be found online in the Supporting Information section

## Figures and Tables

**Figure 1.**
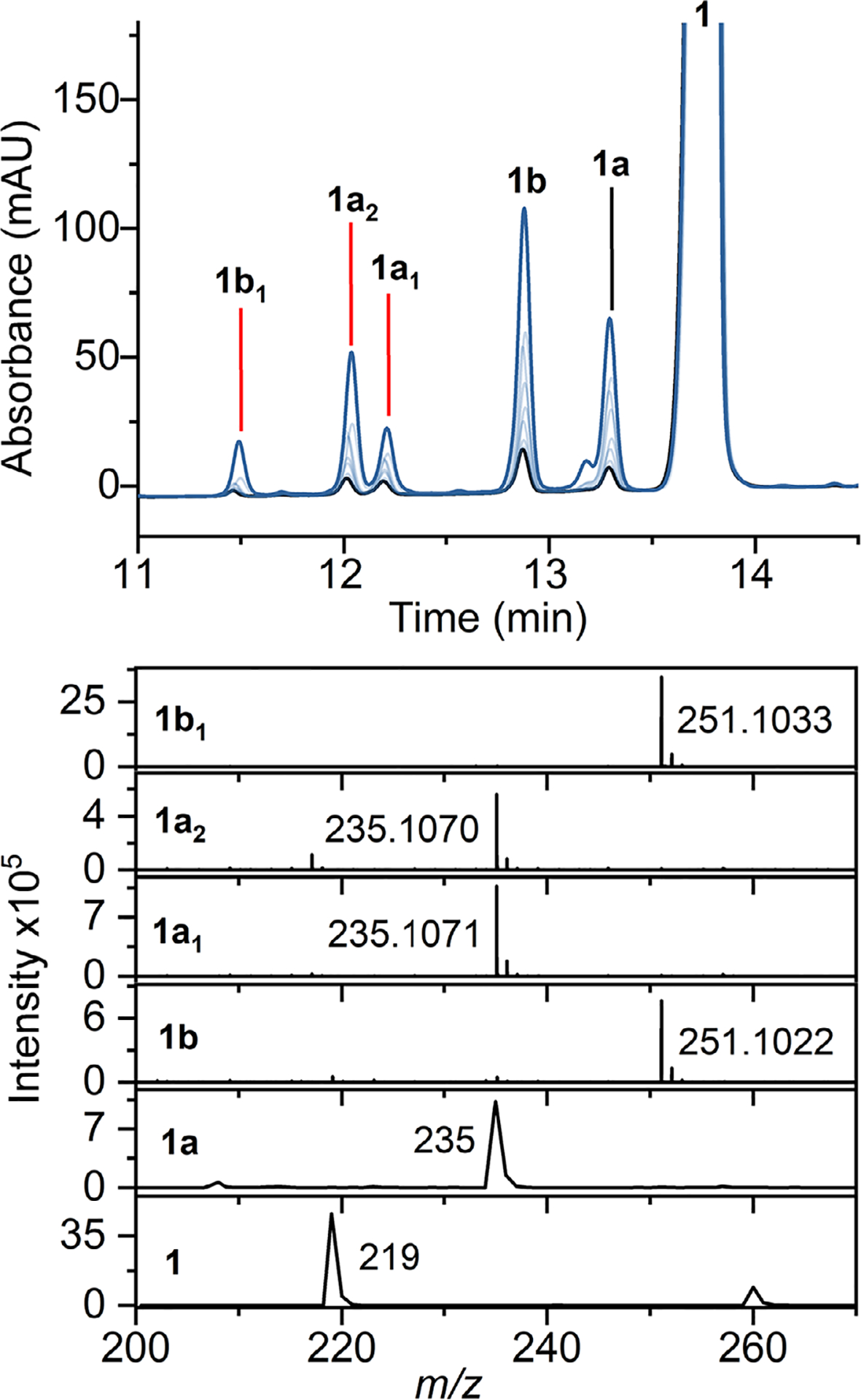
MarE reaction with *β*-Me-l-Trp (**1**) and O_2_. Top panel HPLC time course (10 min: black, 16 h: blue). Bottom panel HRMS analysis. Products: **1a** (2-oxindoline product), **1a**_**1**_, and **1a**_**2**_ (monooxygenated products), **1b** and **1b**_**1**_ (dioxygenated products).

**Figure 2.**
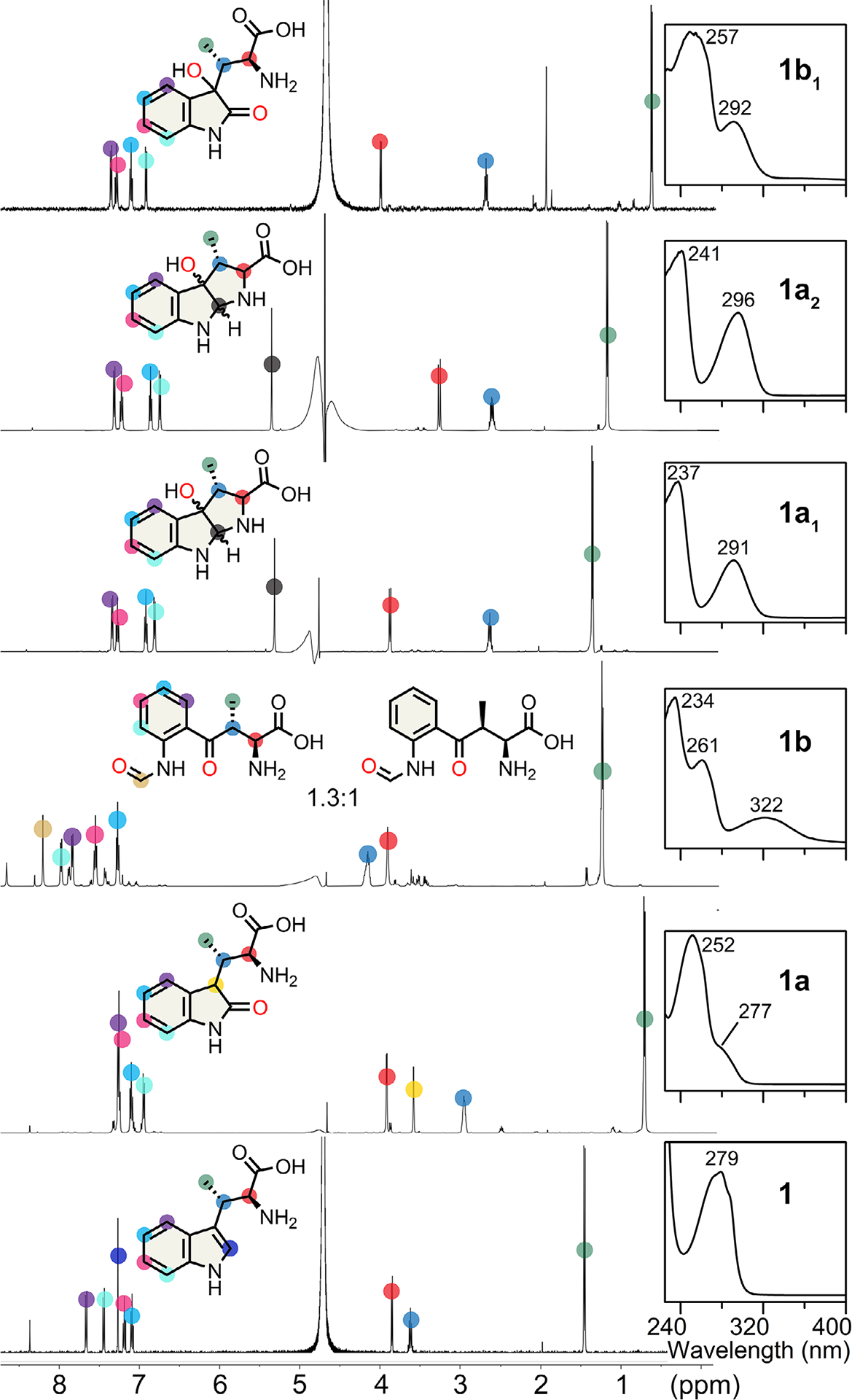
MarE reaction without ascorbate: 2-oxindole, dioxygenation, and pyrroloindoline products of *β*-Me-l-Trp (**1**) characterized by ^1^H-NMR and UV–vis spectroscopies.

**Figure 3.**
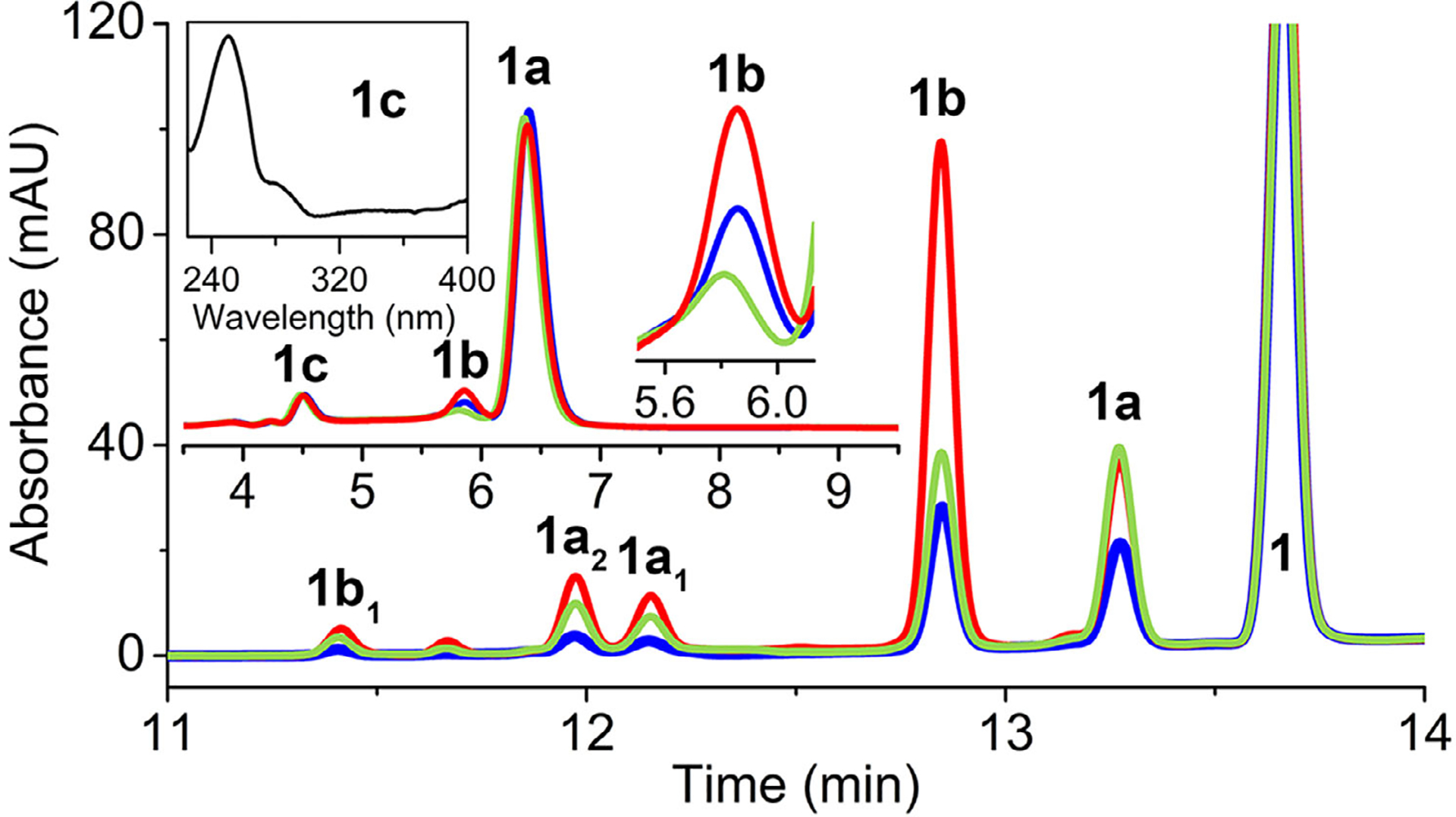
Reaction of **1** in the absence of ascorbate with wild-type MarE (blue), SLGGR-to-SLGGS (light green), and SLGGR-to-GTGGS (red) MarE variants. The insets show the reaction of MarE and its variants in the presence of ascorbate and include the zoomed-in view for peak **1b** and UV–vis spectrum of **1c**.

**Figure 4.**
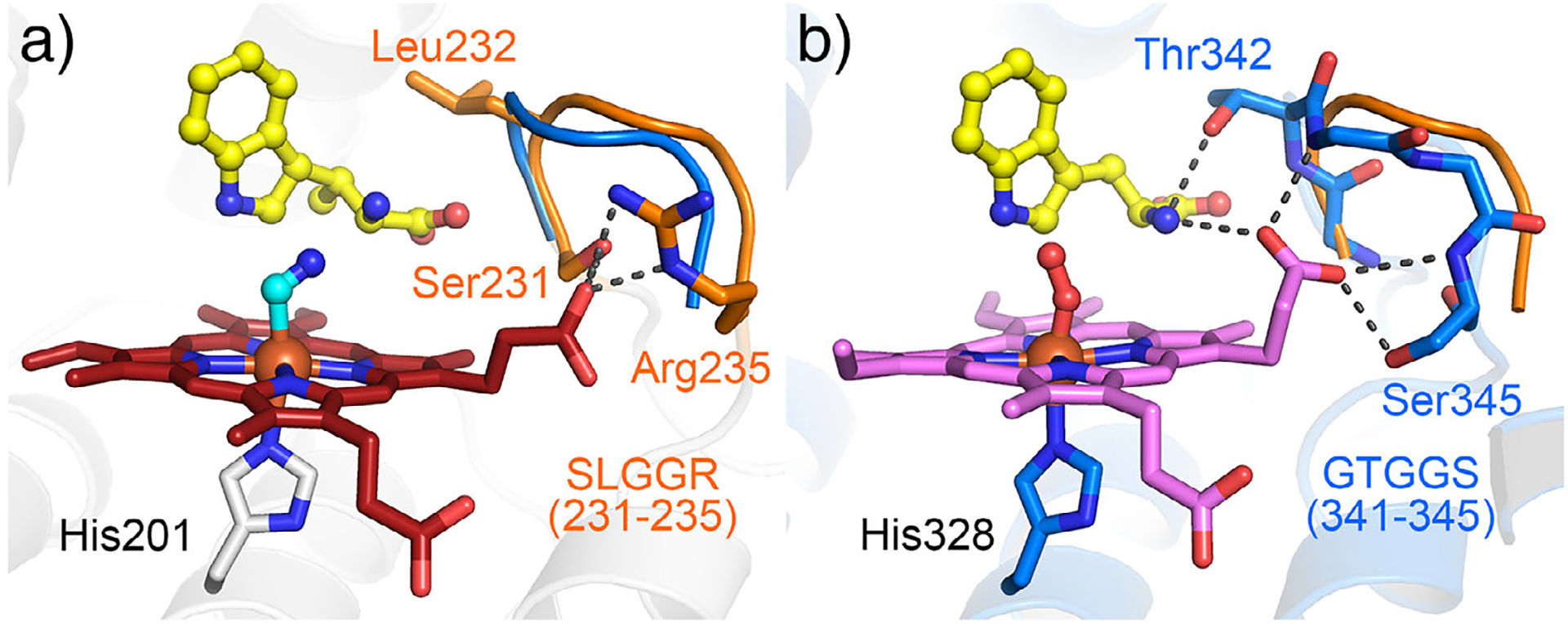
Second coordination sphere comparison. a) MarE (orange, SLGGR loop with *β*-Me-l-Trp in yellow, 9CA3.pdb) versus b) TDO (blue, GTGGS loop with l-Trp/O_2_, 5TI9.pdb).The substrates are shown with their carbons in yellow. The GTGGS loop of TDO (blue) is overlaid with SLGGR loop of MarE (orange).

**Figure 5.**
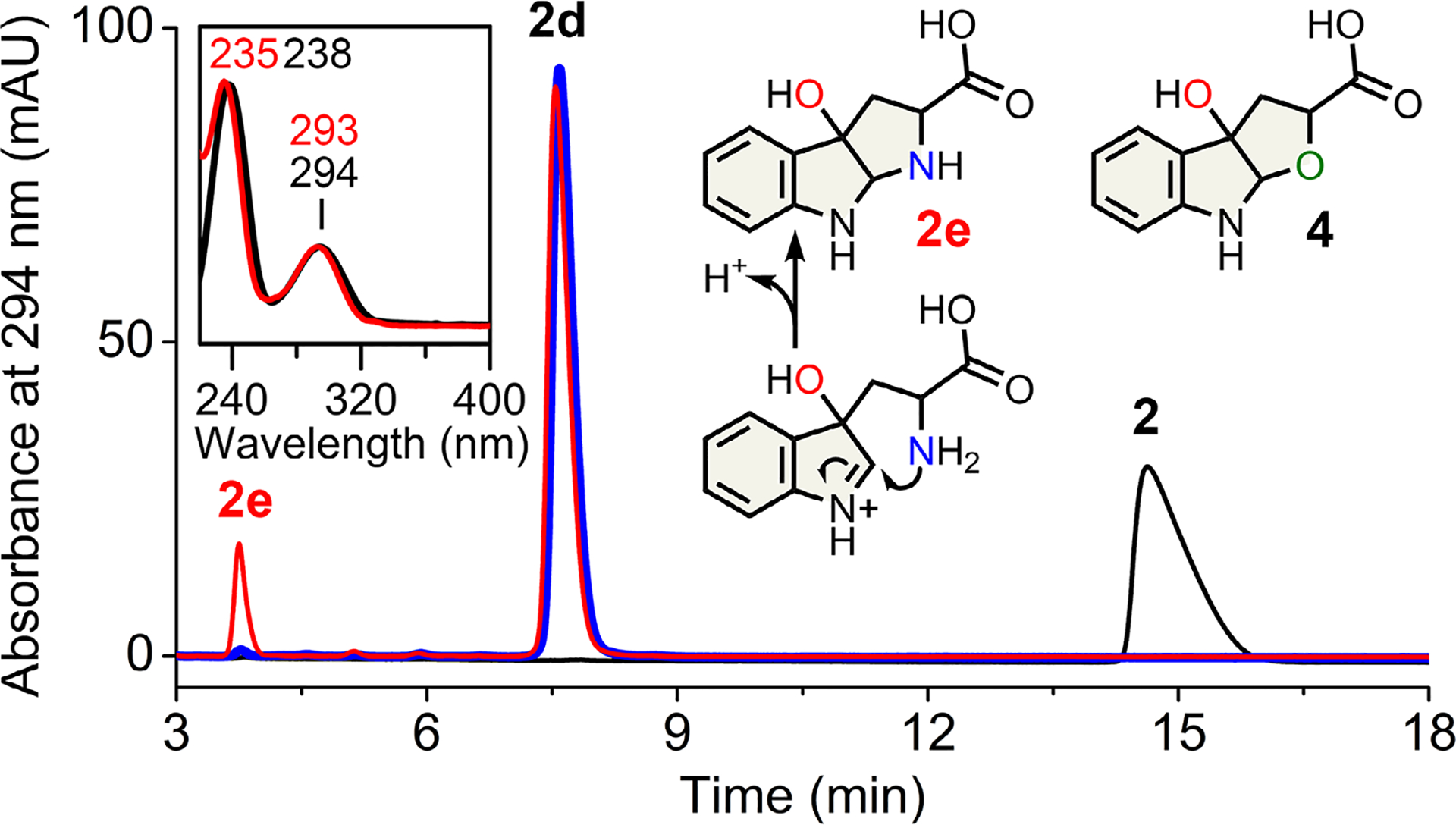
Monooxygenation by GTGGS-to-SLGGS TDO variant. HPLC chromatogram: wild-type (blue) and variant (red). Product **2e** (red) UV–vis versus **4** (black). Structures of **2e** and **4** are shown.

**Scheme 1.**
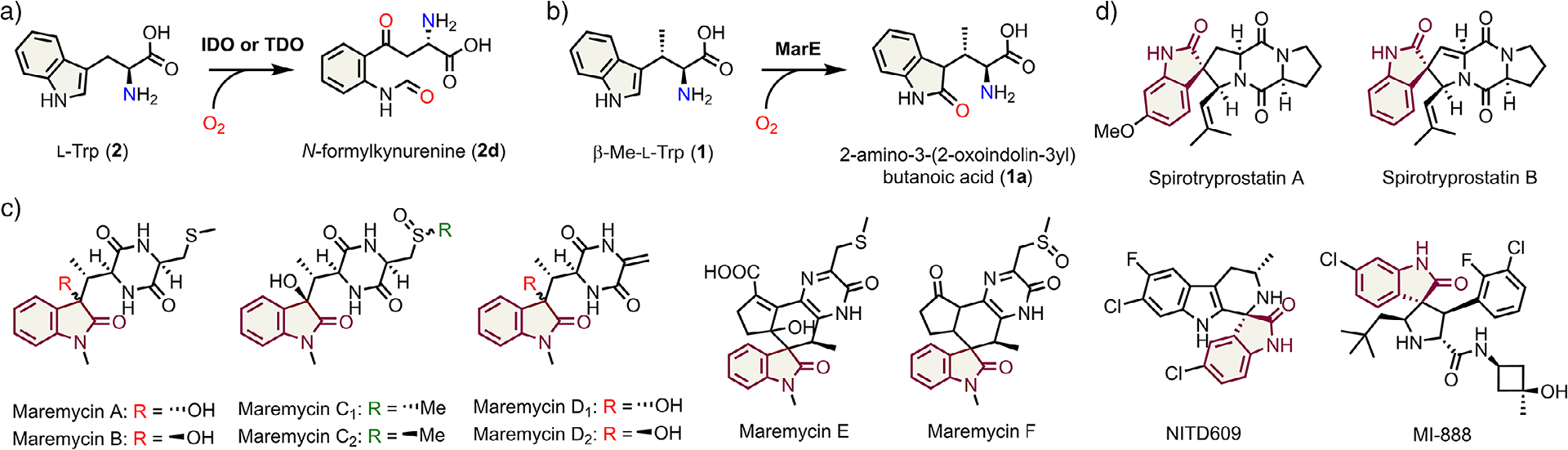
Reactions catalyzed by IDO/TDO a) and MarE b). Naturally occurring maremycins A, B, C1, C2, D1, D2, E, and F c) and spirooxindoles d) share an oxindole structure (maroon color).

**Scheme 2.**
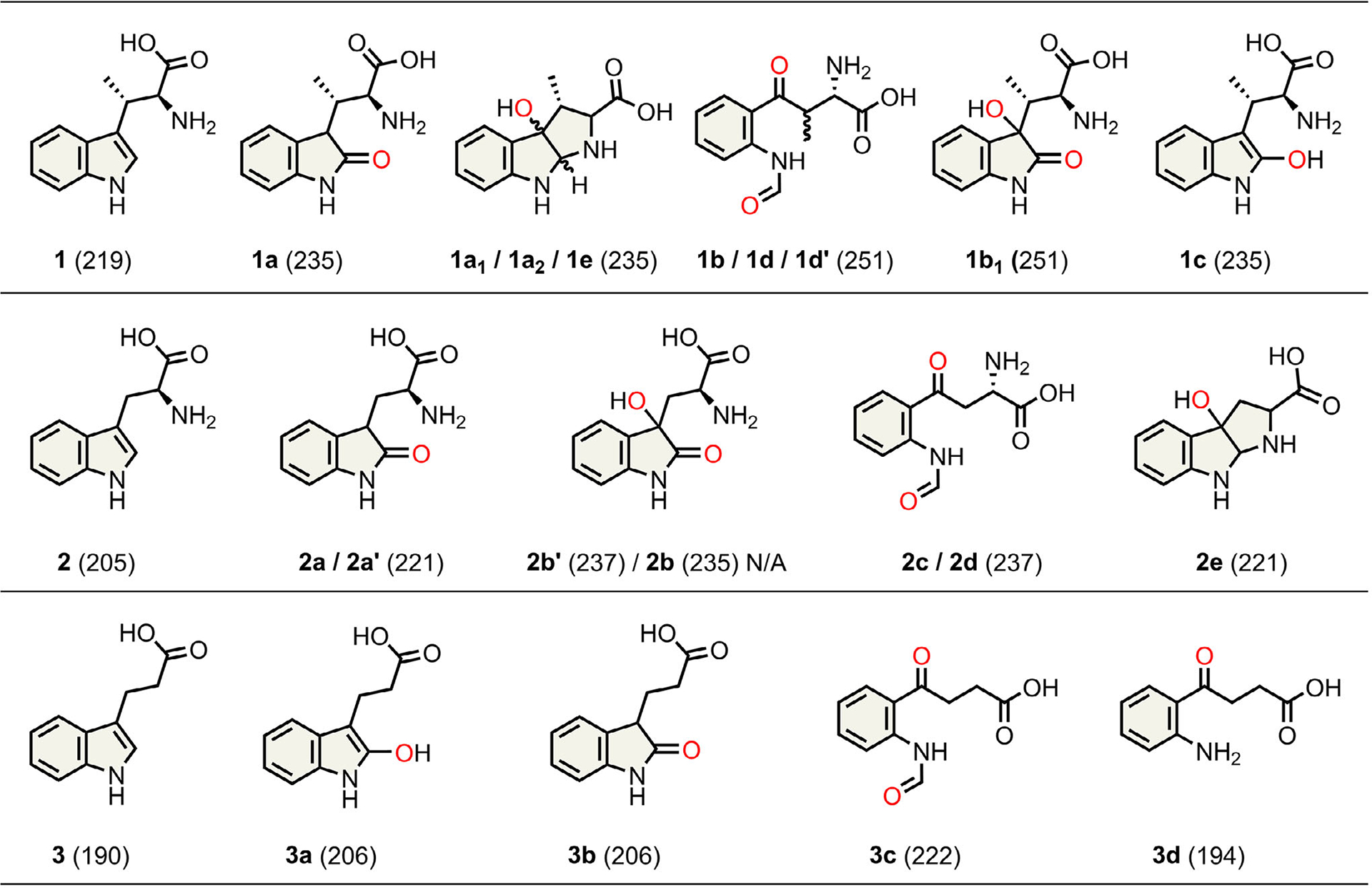
Oxygenation products of the MarE-mediated oxidation reactions identified in this study (*m/z* values in parentheses).

## Data Availability

The data that support the findings of this study are available in the Supporting Information of this article.

## Abbreviations:

*β*-Me-l-Trp: (2*S*,3*S*)-*β*-methyl-l-tryptophan
HDAO: heme-dependent aromatic oxygenase
IDO: indoleamine 2,3-dioxygenase
TDO: tryptophan 2,3-dioxygenase
IPA: indole-3-propionic acid
l-Trp: l-tryptophan
NFK: *N*-formylkynurenine
TyrH: tyrosine hydroxylase
