# Supplementary material for: Deciphering Tryptophan Oxygenation: Key Modulators of 2‐Oxindole Formation in MarE
Source: Angew Chem Int Ed Engl. 2025 Jul 20;64(35):e202510848. doi: 10.1002/anie.202510848 (PMC12312684; doi:10.1002/anie.202510848)
Supplement: Supplementary file 1 — Supporting Information [file ANIE-64-e202510848-s001.docx]

**Supporting Information**

Deciphering Tryptophan Oxygenation: Key Modulators of 2-Oxindole Formation in MarE

Romie C. Nguyen, Inchul Shin*, and Aimin Liu*

Department of Chemistry, The University of Texas at San Antonio, Texas 78249, United States

*To whom correspondence should be addressed:

Aimin Liu (e-Mail: [Feradical@utsa.edu](mailto:Feradical@utsa.edu)) or Inchul Shin (e-Mail: [Inchul.Shin@utsa.edu](mailto:Inchul.Shin@utsa.edu))

**Materials and Methods**

|  | β-Me-L-Trp preparation |
| --- | --- |
|  | Cloning, expression, and purification of MarE |
|  | Heme reconstitution of MarE |
|  | Pyridine hemochromagen assay |
|  | Enzyme reaction |
|  | High-performance liquid chromatography coupled with mass spectrometry (LC-MS) |
|  | High resolution mass spectrometry (HRMS) |
|  | Scaled-up enzyme reaction and product purification for NMR |

**List of Supplementary Tables**

| **Table S1** | The codon-optimized DNA sequence of MarE and primers used in this study for MarE and human TDO mutagenesis studies |
| --- | --- |
| **Table S2** | ^1^H-NMR (500 MHz, D_2_O) and ^13^C-NMR (126 MHz, D_2_O) for MarE product **1b** |
| **Table S3** | ^1^H-NMR (500 MHz, D_2_O) and ^13^C-NMR (126 MHz, D_2_O) for MarE product **1b_1_** |
| **Table S4** | ^1^H-NMR (500 MHz, D_2_O) and ^13^C-NMR (126 MHz, D_2_O) for MarE product **1a_2_** |
| **Table S5** | ^1^H-NMR (500 MHz, D_2_O) and ^13^C-NMR (126 MHz, D_2_O) for MarE product **1a_1_** |

**List of Supplementary Figures**

| **Figure S1** | ^1^H- and ^13^C-NMR of product **1b** in D_2_O from the MarE reaction with dioxygen |
| --- | --- |
| **Figure S2** | ^1^H- and ^13^C-NMR of product **1b_1_** in D_2_O from the MarE reaction with dioxygen |
| **Figure S3** | ^1^H- and ^13^C-NMR of product **1a_2_** in D_2_O from the MarE reaction with dioxygen |
| **Figure S4** | ^1^H- and ^13^C-NMR of product **1a_1_** in D_2_O from the MarE reaction with dioxygen |
| **Figure S5** | Reaction of β-Me-L-Trp in the MarE and variants mediated reaction in the presence of ascorbate |
| **Figure S6**  **Figure S7** | HRMS fragmentation pattern of product **1c** from MarE reaction in the presence of ascorbate  The up-propionate interacts with GTGG motif containing JK-loop in human TDO |
| **Figure S8** | HPLC analysis for the reactions catalyzed by human TDO and variants on IPA (**3**) |
| **Figure S9** | HPLC chromatograms for the reactions catalyzed by TDO and the loop variants on L-Trp (**2**) |
| **Figure S10** | Comparisons of catalytic activities of TDO and the GTGGS-to-SLGGS variant on L-Trp (**2**) |
| **Figure S11** | Comparisons of catalytic activities of TDO and the GTGGS-to-SLGGS variant on the substrate of MarE, β-Me-L-Trp (**1**) |
| **Figure S12** | Near identical catalytic activity of MarE SLGGR-to-GTGGR variant on L-Trp (**2**) compared to wild-type MarE |
| **Figure S13** | The difference in the connectivity of the down-propionate between MarE and TDO |
| **Figure S14** | HPLC chromatograms for the reactions catalyzed by the TDO variants affecting the down- propionate on L-Trp (**2**) |
| **Figure S15** | HPLC analysis for the reaction products of IPA (**3**) as an alternative substrate |
| **Figure S16** | HPLC analysis for the reactions catalyzed by MarE and its loop variants on IPA (**3**) |
| **Figure S17** | The product formation profiles between **3b** and **3c** in the reactions promoted by human TDO, MarE, and their variants |

**References cited**

**Materials and Methods**

**β-Me-L-Trp preparation**

Synthesis of β-Me-L-Trp (**1**) followed the previously described method.^[1]^ The Francis Arnold Lab generously shared the *Pf*TrpB^2B9^ expression vector with us. Cell culture, gene expression, and protein purification were conducted as described. The purified *Pf*TrpB^2B9^ was concentrated to 650 μM and stored at -80°C until use. In summary, indole (63.2 mg, 0.54 mmol) and L-threonine (595.8 mg, 5 mmol) were placed in a 20 mL vial. The solid mixture was then suspended in 3.5 mL of a 5% DMSO buffer solution containing 200 mM potassium phosphate (pH 8.0). A 15 mM stock solution of pyridoxal phosphate was prepared in water and subsequently added to the mixture to achieve a final concentration of 100 μM. Purified *Pf*TrpB^2B9^ was added to a final concentration of 160 μM in a 5 mL reaction vial, then heated to 75°C in an oil bath while stirring. Following 18 h of reaction, the mixture was removed from heat, allowed to cool to room temperature, and the solids were subsequently pelleted via centrifugation. The crude supernatant was subjected to purification using a Teledyne ISCO Combiflash Rf system, which was equipped with a RediSep Gold C18 column. The column was washed with 2 column volumes (CVs) of water prior to performing an isocratic elution using a 50% methanol/water mixture. The solvent was subsequently removed under reduced pressure, resulting in the recovery of a lightly tan solid with a yield of 63.4%. Refer to Figure 2 in the main text for ^1^H-NMR spectra of the purified β-Me-L-Trp (1). ^1^H-NMR (300 MHz, D_2_O) δ 7.67 (dt, *J* = 8.0, 1.1 Hz, 1H), 7.45 (dt, *J* = 8.2, 1.0 Hz, 1H), 7.27 (s, 1H), 7.19 (ddd, *J* = 8.2, 7.0, 1.3 Hz, 1H), 7.09 (ddd, *J* = 8.1, 7.0, 1.2 Hz, 1H), 3.86 (d, *J* = 6.6 Hz, 1H), 3.63 (p, *J* = 7.1 Hz, 1H), 1.46 (d, *J* = 7.3 Hz, 3H).

**Cloning, expression, and purification of MarE**

A codon-optimized gene encoding the full-length MarE protein (amino acids 1 – 284) from *Streptomyces* sp. B9173 (UniProt ID: X2D878) was synthesized and obtained from GenScript. The synthesized MarE gene, located between NdeI and HindIII sites, was cloned into a modified pET-28a vector (Merck), named pET-28aTEV. This vector contains a Tobacco etch virus (TEV) protease cleavage site, which is intended for the removal of the N-terminal polyhistidine tag. The DNA sequences of the constructed expression vector plasmids were confirmed through DNA sequencing conducted by Eurofins Genomics.

MarE proteins with His-tags were produced in *E. coli* BL21 (DE3). Cells were cultured in Luria Bertani (LB) medium at 37°C with shaking at 220 rpm. The expression of the MarE gene was induced by adding 0.5 mM isopropyl-L-thio-β-D-galactopyranoside (IPTG) once the optical density at 600 nm (OD600) reached 0.8. The temperature was reduced to 20 °C, and the cells were cultured for an additional 16 to 20 h.

The harvested cells were resuspended in a solution of buffer A, which consists of 50 mM Tris-HCl, 200 mM NaCl, and 5% glycerol (pH 8.0) per gram of wet biomass. The cell membrane was disrupted using a sonicator (Thermo Fisher Scientific). A 300 mL cell suspension was stirred on wet ice with a 1 h pulse cycle of 1 sec on/1 sec off. Cell debris was removed by centrifugation at 34,000 x g for 1 h at 4°C.

The supernatant containing His‐tagged MarE was subsequently purified using immobilized metal affinity chromatography (IMAC) with a HisTrap column (Cytiva), pre-equilibrated with buffer A. The His-tagged protein was eluted using buffer B, which consists of buffer A and 500 mM imidazole. Histag removal was performed by treating the IMAC elution fraction with TEV protease during dialysis in buffer C, which contained 50 mM Tris-HCl, 50 mM NaCl, and 10 mM β-mercaptoethanol (pH 7.5) at 4 °C overnight. The tag-removed MarE protein was separated and collected using a HisTrap column with buffer A/B. The flow-through fraction was concentrated and desalted with a HiTrap Desalting column (Cytiva) in 50 mM HEPES-NaOH, 50 mM NaCl, 5% glycerol (pH 7.5). This buffer-exchanged, untagged MarE protein was used for heme reconstitution. The extinction coefficient at 280 nm (ε_280_) for the untagged full-length MarE protein was calculated as 46,410 M^-1^cm^-1^ with a molecular weight of 31,996.77 Da using the Expasy ProtParam tool (https://web.expasy.org/protparam).

**Heme reconstitution of MarE**

A 4 mM stock solution of hemin chloride was freshly prepared by dissolving the compound in 50 mM NaOH. Hemin chloride, at a final concentration of 60 μM, was added to 50 μM of untagged MarE protein to achieve a hemin to protein ratio of 1.2:1 with gentle stirring. The reconstitution reaction was performed at room temperature for 3 h, followed by overnight incubation at 4°C. The reaction mixture was then centrifuged at 34,000 x g for 10 min at 4°C to remove precipitates. The supernatant was concentrated and desalted into a buffer containing 50 mM HEPES, 50 mM NaCl, and 5% glycerol (pH 7.5) to eliminate excess hemin. The heme-reconstituted protein was further concentrated and stored at -80°C. Through heme reconstitution, MarE achieved a heme occupancy of at least 70%, as determined by the heme extinction coefficient at 405 nm (ε_405_ = 169,198 M^-1^cm^-1^) using the pyridine hemochromagen method.

**Pyridine hemochromagen assay**

The quantity of heme was measured with the pyridine hemochromagen assay, which has an extinction coefficient (ε_557_) of 34 mM^-1^cm^-1^ for its reduced form.^[2]^

**Enzyme reaction**

Enzyme reactions were prepared with the following components: 50 μM enzyme (heme), 20 mM or 1 mM sodium ascorbate, and 1 mM substrate (β‐Me-L-Trp (**1**), L-Trp (**2**), and IPA (**3**)) in Buffer A, which consists of 50 mM Tris-HCl, 200 mM NaCl, and 5% glycerol (pH 8.0). The reactions were conducted at a volume of 250 μL at room temperature for a duration of 16 h. Termination of the reactions involved filtration using a 10 kDa molecular weight cut-off (MWCO) centrifugal filter (Merck Millipore).

**High-performance liquid chromatography (HPLC) coupled with mass spectrometry (LC-MS)**

The use of a full wavelength detector in HPLC in our studies, including this and previous work,^[3]^ reporting the full UV-vis spectrum of each reaction product, instead of a single-wavelength detector without the full spectrum of the reaction products,^[4]^ was ultimately important in providing a complete description of the MarE reaction.

Each filtered reaction mixture was examined using a rapid separation system, specifically employing the Ultimate-3000SD HPLC from Thermo Fisher Scientific, which featured both a photodiode array detector and an ISQ EC mass spectrometer. For the analysis, 20 μL of each sample was injected into an InertSustain C18 column with a particle size of 5 μm and dimensions of 4.6 I.D. x 100 mm by GL Sciences Inc. The elution process was conducted isocratically with a solvent blend of water, acetonitrile, and 0.1% formic acid at a flow rate of 1.0 mL/min. Due to varying solubilities, the following acetonitrile concentrations were utilized: 6.0% for β-Me-L-Trp (**1**), 2.7% for L-Trp (**2**), and 20% for IPA (**3**). Each elution run lasted 20 min.

**High resolution mass spectrometry (HRMS)**

High-resolution mass spectra were acquired using a maXis plus quadrupole-time of flight mass spectrometer equipped with an electrospray ionization source (Bruker Daltonics) in positive ionization mode. Liquid chromatography fraction samples were introduced at a flow rate of 3 μL/min via a syringe pump. Key parameters for the ion source were set as follows: capillary voltage at 3500 V, endplate offset at –500 V, nebulizer gas pressure at 0.4 bar, dry gas flow at 4.0 L/min, and source temperature at 200°C. Mass spectra were recorded at one scan per second over a range of 50-1500 *m*/*z*, and averaged over one minute. The Compass Data Analysis software version 4.3 (Bruker Daltonics) was used to process all mass spectra.

**Scaled-up enzyme reaction and product purification for NMR**

In order to accumulate sufficient product for NMR analysis of the dioxygenated products of β‐Me-L-Trp (**1**), 15 - 25 mL reactions were carried out with 180 μM MarE reduced with 1.1 equivalents of dithionite prepared in anaerobic buffer using the Schlenk line and gas-tight Hamilton syringes. The substrate β‐Me-L-Trp (**1**) was dissolved in oxygen-saturated buffer to a final 2 mM concentration and titrated 1 mL at a time into the reduced MarE mixture while gently stirring the mixture on ice. The resulting reaction mixture was allowed to proceed at 4°C. After 16 h, the enzyme was removed using a 10 kDa filter, and the filtrate was lyophilized to a beige, white solid to prepare for purification. Approximately 320 mL of reaction was needed to generate a sufficient amount of each product for NMR analysis.

**NMR analysis of the reaction products collected via preparative HPLC and lyophilization**

A separate semi-preparative Ultimate-3200BX HPLC with a multiple wavelength MWD-3000 detector from Thermo Fisher Scientific was used to repeatedly pool the reaction products for NMR analyses. A Labconco Lyophilizer System, FreeZone 2.5 Liter -84 °C Benchtop Freeze Dryer, was used to aid the sample preparation.

The white solid was dissolved in 3 mL nanopure water and injected into the semi-preparative HPLC equipped with a 250 mm x 20 mm internal diameter column (Thermo Fisher Scientific) and a fraction collector. The fractions for each product were collected in separate 50 mL conical tubes and lyophilized to prepare for purification on the analytical Ultimate‐3000SD HPLC rapid separation system equipped with a photodiode array detector. Each product fraction was dissolved in 500 μL nanopure water and injected into the analytical HPLC. Each product was collected in 15 mL conical tubes and lyophilized. NMR samples were made in D_2_O using thick-walled NMR tubes and analyzed on a Bruker 500 MHz Avance III equipped with a 5 mm Prodigy CryoProbe.

NMR spectra were acquired on a Bruker 500 MHz Avance III HD spectrometer operating at 11.7 T. TopSpin 3.5pl6 was used for data processing.

**Table S1**. The codon-optimized DNA sequence of MarE and primers used in this study for MarE and human TDO mutagenesis studies

| Codon-optimized DNA sequence of MarE | | |
| --- | --- | --- |
| ATGAAGCGTAGCCTGAACCCGGACGAACCGAACGCGCTGCTGAGCTACGACTTCGATCGTGGCAGCAACT  ATGAGAACGTGCTGCATCTGACCGATGCGCTGGGTGCGCTGGTTCCGGAGAGCGAAACCGAGCACCCGGA  TCAGCGTTTCTTTCAAGTTACCCACCTGATCACCGAATACGCGTGGGTGCAGGTTCACTATGAGCTGCGT  CGTGCGATTGGCCACCTGGACGAAGATCGTTACCACCAAGCGGTTCGTATGTTTGACCGTGCGACCGGTC  TGAGCGAGGTGACCGTTCAGGCGGTGCGTCTGCTGACCGATCACCTGCCGCAACACAGCCTGCTGATGAT  GCGTAACGCGCTGCCGGAAGATGCGACCGGTCTGGATAGCCCGGGTTACCGTAACCTGCGTCGTGTGGCG  CGTCCGGTTTGGAAAGCGTATGAACAAGCGGTGGAGCGTGCGGGTCTGAGCCTGCAAGACGTTATCGCGC  AGCAAGACGATGGCTATGATGGTCCGCGTAGCGGTGGCAGCCAGAGCCTGGCGCTGGTGCGTGAGGCGAT  GCTGCGTCTGGACGGCAGCGTTCTGGGTTGGAAGCAACACCACCTGATTATGGTGTGGAGCCAGCTGGGT  GGCCAACCGGGTCTGCGTAAGGGTAACGAGGAAGGCGACGATGGTCTGGAACTGCCGCAGAGCCTGGGTG  GCCGTAGCCTGGCGACCCTGGAGGCGCGTAGCCAACTGGCGCTGTTTCCGGAACTGTGGCGTGCGGCGGA  GGATGCGTACTGGCTGCTGGGTACCCGTCATGACACCGATGCGCCGGTGCGTGGTGGCGGTAACGGTTGC  CCGGTTCAGCACTAA | | |
| Mutagenesis primers | | |
|  | MarE L232T | AACTGCCGCAGAGCACGGGTGGCCGTAGCC |
|  | MarE S231G on L232T | AACTGCCGCAGGGCACGGGTGGC |
|  | MarE R235S (SLGGR-to-SLGGS) | AGCCTGGGTGGCTCTAGCCTGGCGAC |
|  | MarE R235S on SLGGR-to-GTGGR | GCACGGGTGGCTCTAGCCTGGCGA |
|  | TDO GTGGS-to-SLGGS | TGGGCAGCAAAGCTAGCCTCGGTGGTTCCTCA |
|  | TDO GTGGS-to-GTGGA | TGGCACCGGTGGTGCCTCAGGCTATCA |
|  | TDO GTGGS-to-GPPGS | AGCAAAGCTGGCCCCCCTGGTTCCTCAGGC |
|  | TDO GTGGS-to-G_GGS | AGCAAAGCTGGC___GGTGGTTCCTCA |
|  | TDO R159A | TCCAGAGTTTGCAATTCGCACTATTAGAAAACAAGA |
|  | TDO Y350F | TTCCTCAGGCTATCACTTCCTGCGATCAACTGTGA |
|  | TDO R325A | ACTGATGACCAAATGGGCATATAACCATGTGTGC |

The forward primers for mutagenesis are provided, and the reverse primers are the reverse complement of these forward primers. Site-directed mutagenesis was conducted employing the QuickChange PCR protocol (Agilent Technologies).

**Table S2**. ^1^H-NMR (500 MHz, D_2_O) and ^13^C-NMR (126 MHz, D_2_O) for MarE product **1b**.

| Label | δ_H_ | | δ_C_ |
| --- | --- | --- | --- |
|  |  |  | |
| 1' |  | 163.07 | |
| 2' | 3.93 (d, *J* = 4.8 Hz, 1H) | 56.42 | |
| 3' | 4.19 (td, *J* = 7.3, 4.5 Hz, 1H) | 42.65 | |
| 4' | 1.25 (d, *J* = 7.1 Hz, 3H) | 13.25 | |
| 1 |  |  | |
| 2 |  | 206.40 | |
| 3 |  | 165.53 | |
| 3a |  |  | |
| 4 | 8.03 (d, *J* = 8.3 Hz, 1H) | 123.88 | |
| 5 | 7.32 (t, *J* = 7.7 Hz, 1H) | 125.49 | |
| 6 | 7.60 (t, *J* = 7.8 Hz, 1H) | 134.40 | |
| 7 | 7.88 (d, *J* = 7.9 Hz, 1H) | 130.17 | |
| 7a |  |  | |

**Table S3**. ^1^H-NMR (500 MHz, D_2_O) and ^13^C-NMR (126 MHz, D_2_O) for MarE product **1b_1_**.

| Label | δ_H_ | | δ_C_ |
| --- | --- | --- | --- |
|  |  |  | |
| 1' |  |  | |
| 2' | 4.03 (d, *J* = 7.0 Hz, 1H) | 56.52 | |
| 3' | 2.73 (p, *J* = 7.2 Hz, 1H) | 40.10 | |
| 4' | 0.67 (d, *J* = 7.3 Hz, 3H) | 11.59 | |
| 1 |  |  | |
| 2 |  |  | |
| 3 |  |  | |
| 3a |  |  | |
| 4 | 7.38 (d, *J* = 7.5 Hz, 1H) | 123.83 | |
| 5 | 7.13 (t, *J* = 7.6 Hz, 1H) | 123.67 | |
| 6 | 7.31 (t, *J* = 7.7 Hz, 1H) | 130.41 | |
| 7 | 6.94 (d, *J* = 8.2 Hz, 1H) | 110.90 | |
| 7a |  |  | |

**Table S4**. ^1^H-NMR (500 MHz, D_2_O) and ^13^C-NMR (126 MHz, D_2_O) for MarE product **1a_2_**.

| Label | δ_H_ | | δ_C_ |
| --- | --- | --- | --- |
|  |  |  | |
| 1' |  | 172.34 | |
| 2' | 3.27 (d, *J* = 12.3 Hz, 1H) | 64.26 | |
| 3' | 2.62 (dq, *J* = 13.5, 6.9 Hz, 1H) | 47.13 | |
| 4' | 1.19 (d, *J* = 6.9 Hz, 3H) | 11.50 | |
| 1 |  |  | |
| 2 | 5.37 (s, 1H) | 82.93 | |
| 3 |  | 90.15 | |
| 3a |  | 125.51 | |
| 4 | 7.33 (d, *J* = 7.6 Hz, 1H) | 126.61 | |
| 5 | 6.88 (t, *J* = 7.5 Hz, 1H) | 131.01 | |
| 6 | 7.24 (t, *J* = 7.7 Hz, 1H) | 120.04 | |
| 7 | 6.76 (d, *J* = 8.0 Hz, 1H) | 110.85 | |
| 7a |  | 149.71 | |

**Table S5**. ^1^H-NMR (500 MHz, D_2_O) and ^13^C-NMR (126 MHz, D_2_O) for MarE product **1a_1_**.

| Label | δ_H_ | | δ_C_ |
| --- | --- | --- | --- |
|  |  |  | |
| 1' |  | 172.24 | |
| 2' | 3.82 (d, *J* = 8.6 Hz, 1H) | 66.08 | |
| 3' | 2.58 (p, *J* = 7.3 Hz, 1H) | 45.52 | |
| 4' | 1.30 (d, *J* = 7.1 Hz, 3H) | 11.45 | |
| 1 |  |  | |
| 2 | 5.26 (s, 1H) | 84.13 | |
| 3 |  | 88.27 | |
| 3a |  | 129.52 | |
| 4 | 7.29 (d, *J* = 7.5 Hz, 1H) | 123.22 | |
| 5 | 6.86 (t, *J* = 7.5 Hz, 1H) | 120.77 | |
| 6 | 7.22 (t, *J* = 7.7 Hz, 1H) | 130.80 | |
| 7 | 6.75 (d, *J* = 8.0 Hz, 1H) | 111.41 | |
| 7a |  | 147.59 | |


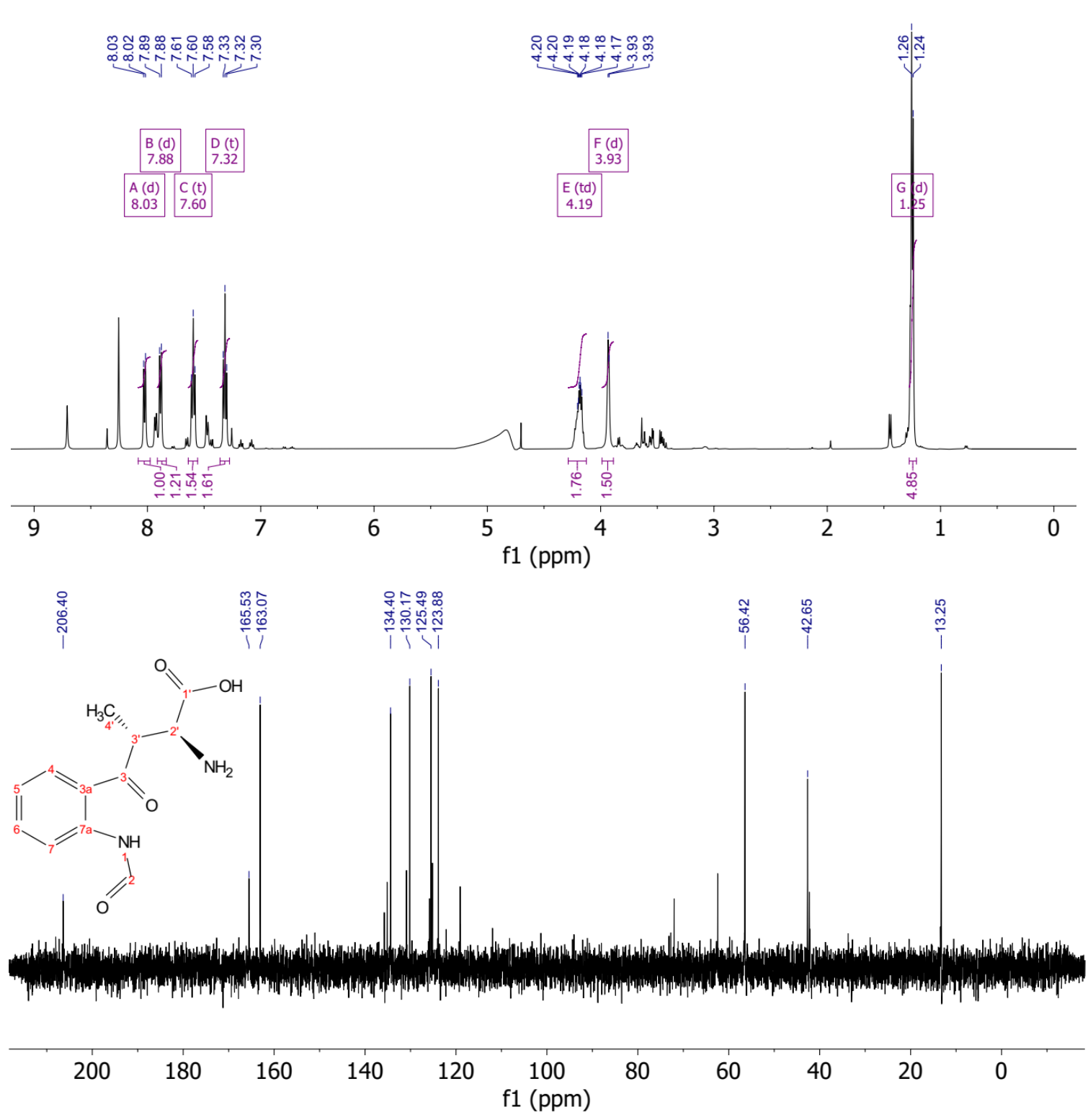


**Figure S1**. ^1^H- and ^13^C-NMR of product **1b** in D_2_O from the MarE reaction with dioxygen.


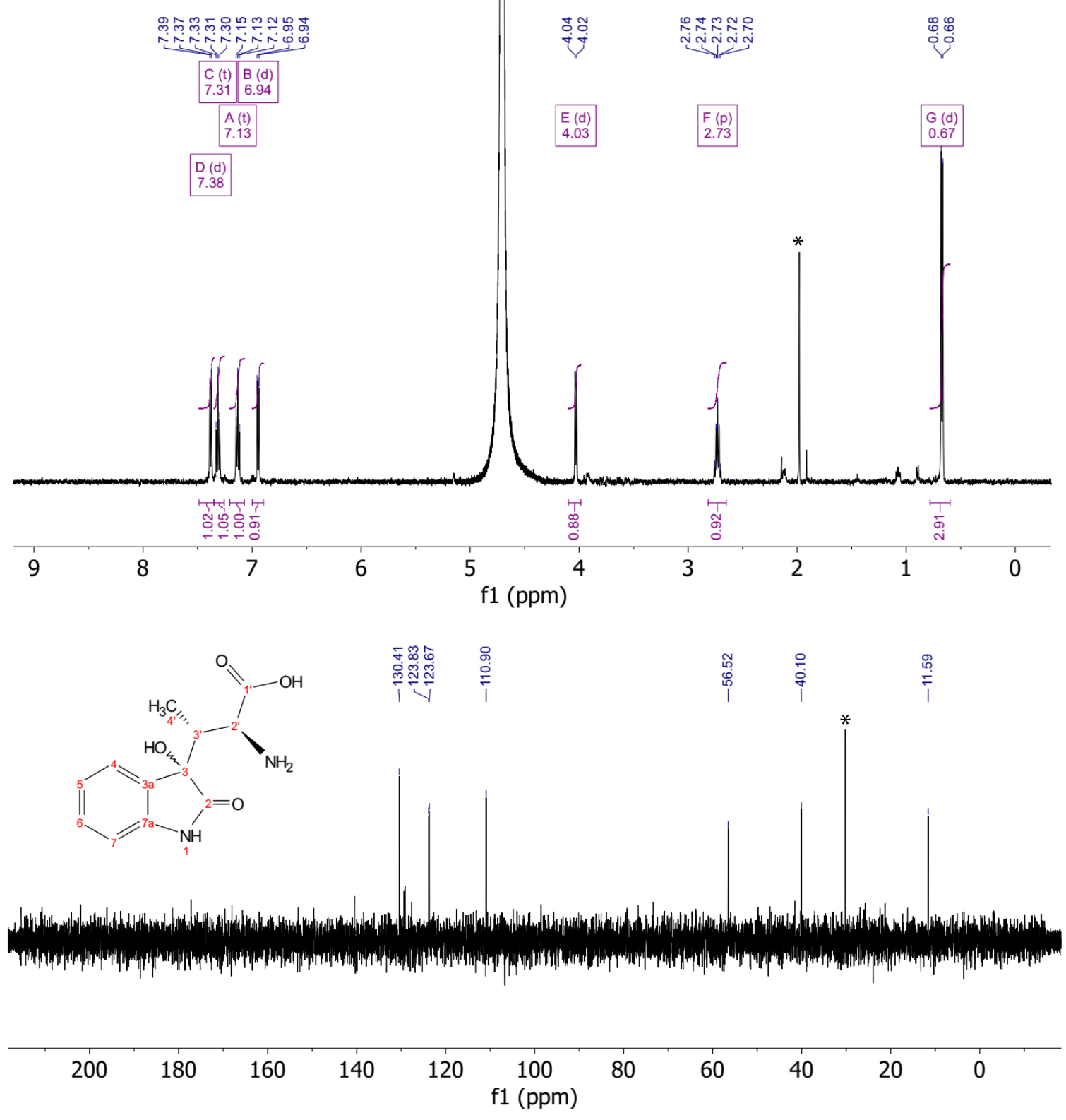


**Figure S2.** ^1^H- and ^13^C-NMR of product **1b_1_** in D_2_O from the MarE reaction with dioxygen (* denotes an impurity not associated with the structure of the product). The quaternary carbons are not resolved due to low S/N.


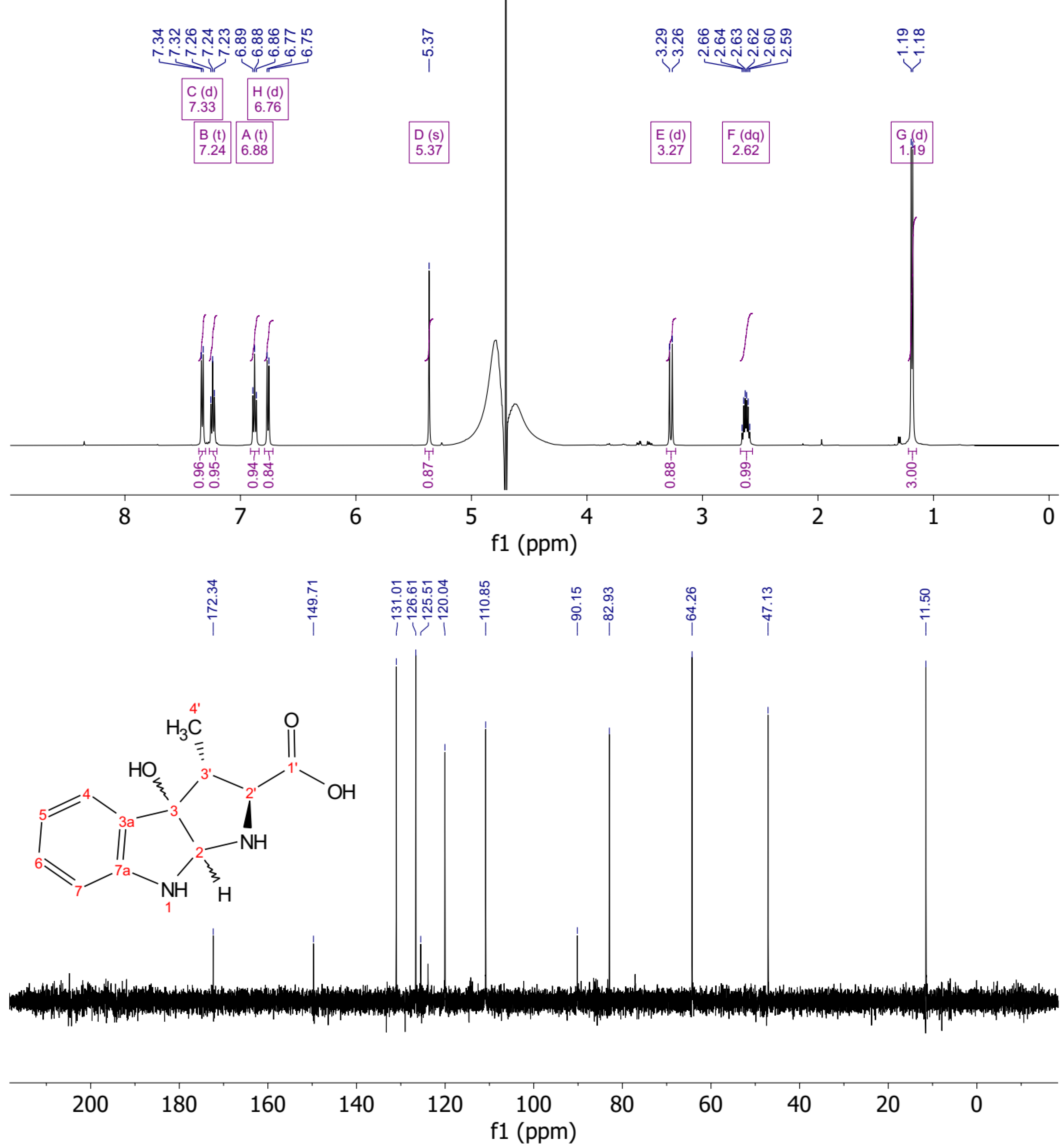


**Figure S3.** ^1^H- and ^13^C-NMR of product **1a_2_** in D_2_O from the MarE reaction with dioxygen.


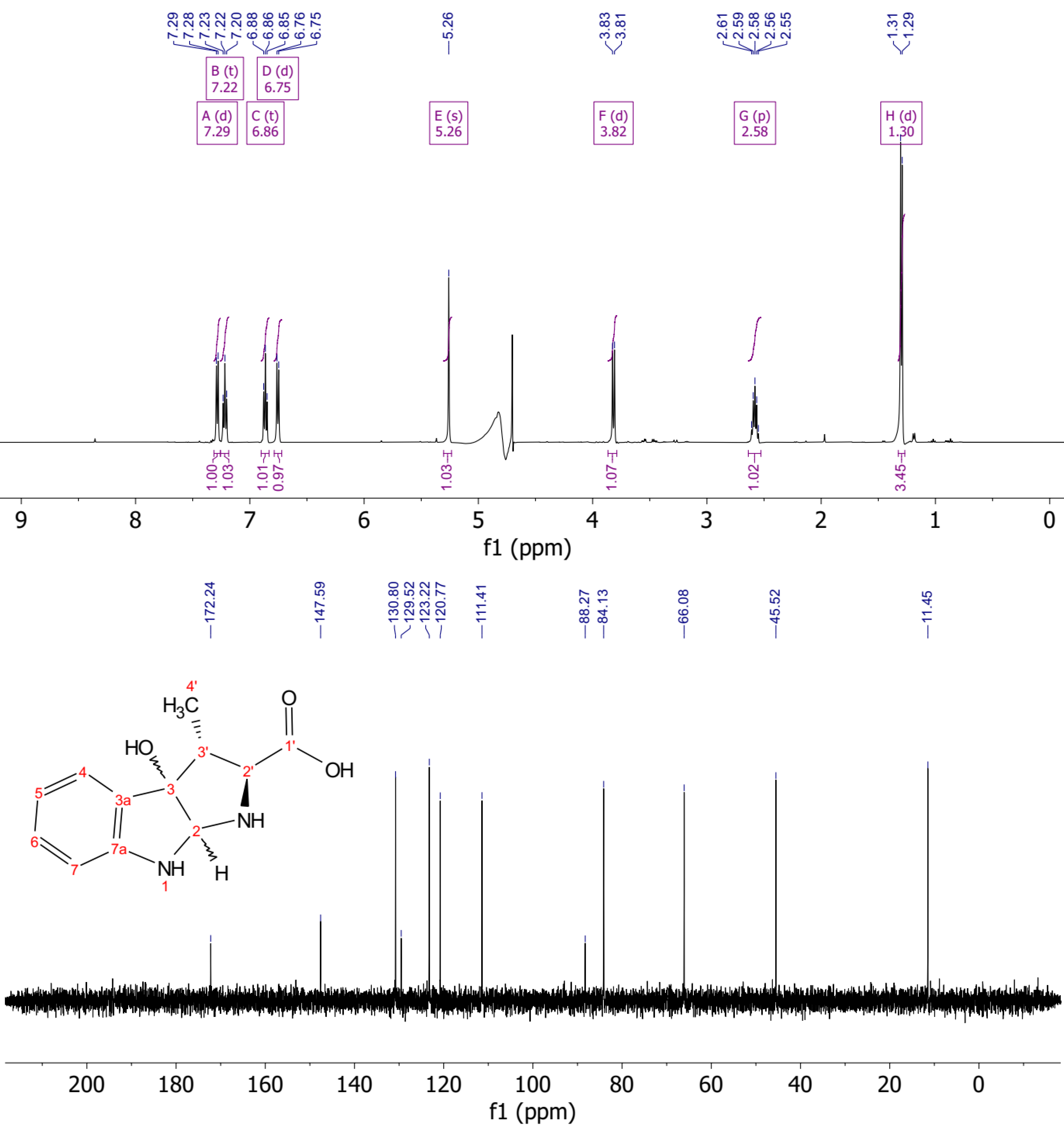


**Figure S4.** ^1^H- and ^13^C-NMR of product **1a_1_** in D_2_O from the MarE reaction with dioxygen.


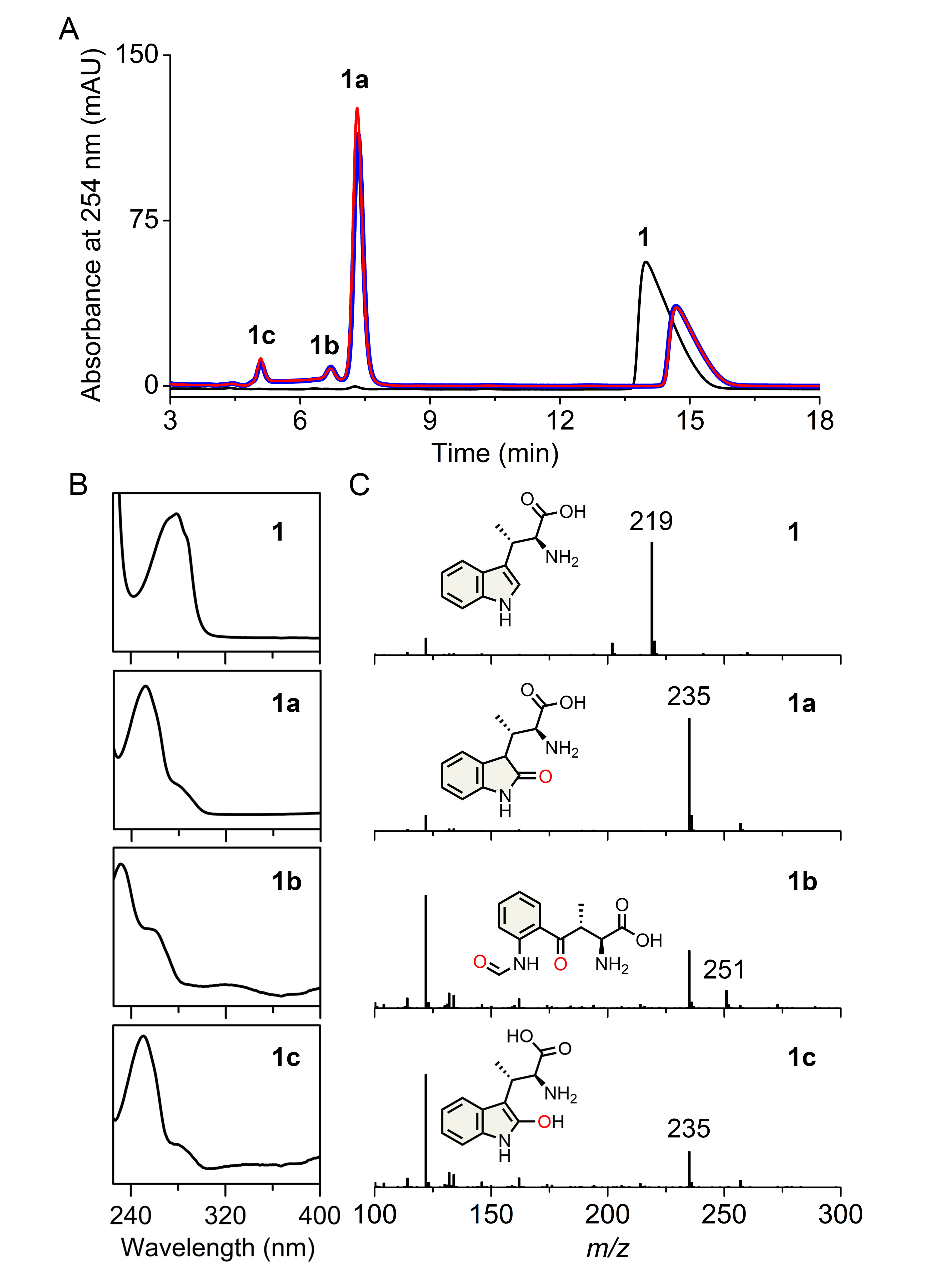


**Figure S5**. Reaction of β-Me-L-Trp in the MarE and variants mediated reaction in the presence of ascorbate. (A) The reactions catalyzed by MarE wild-type (blue trace) and SLGGR-to-GTGGR variant (red trace) with β-Me-L-Trp (**1,** black trace) and ascorbate in the presence of O_2_ were analyzed by HPLC. Reactions were carried out using enzyme (50 μM heme), ascorbate (20 mM), and β-Me-L-Trp (1 mM). (B) UV-vis spectra of substrate **1** and products **1a**, **1b**, and **1c**. (C) Mass spectra of the peaks from the HPLC elution profile and their chemical structures.


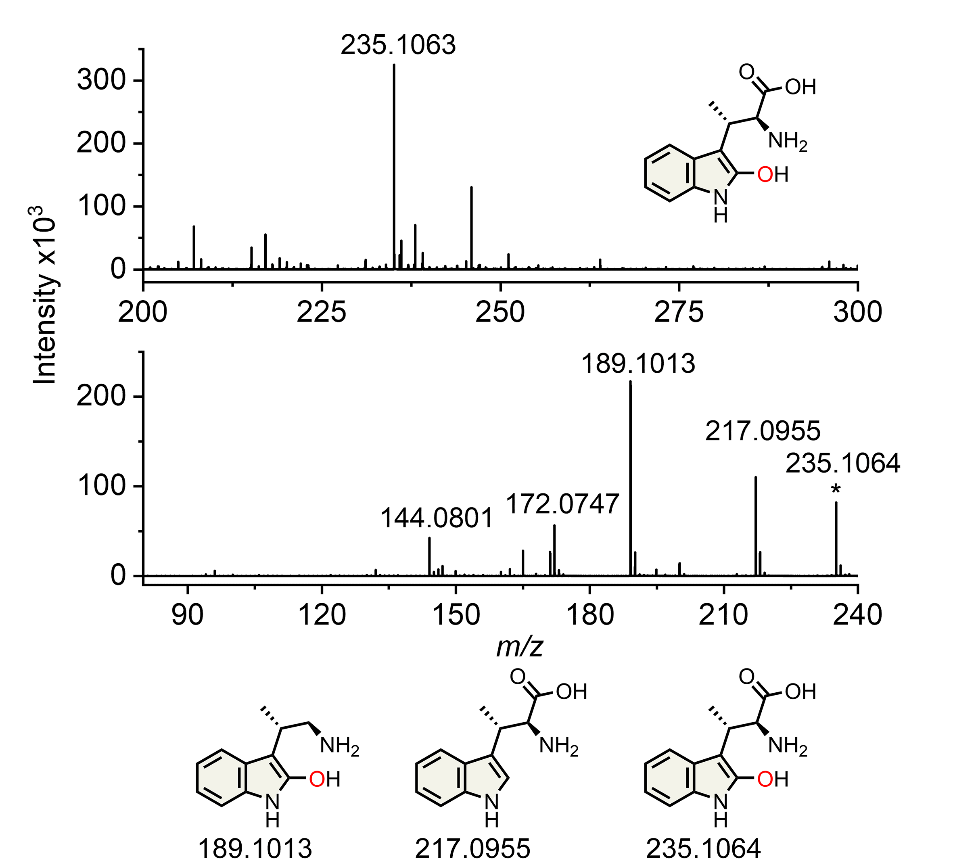


**Figure S6**. HRMS fragmentation pattern of product **1c** from MarE reaction in the presence of ascorbate. Asterisk mark (*) denotes the parent ion for fragmentation.


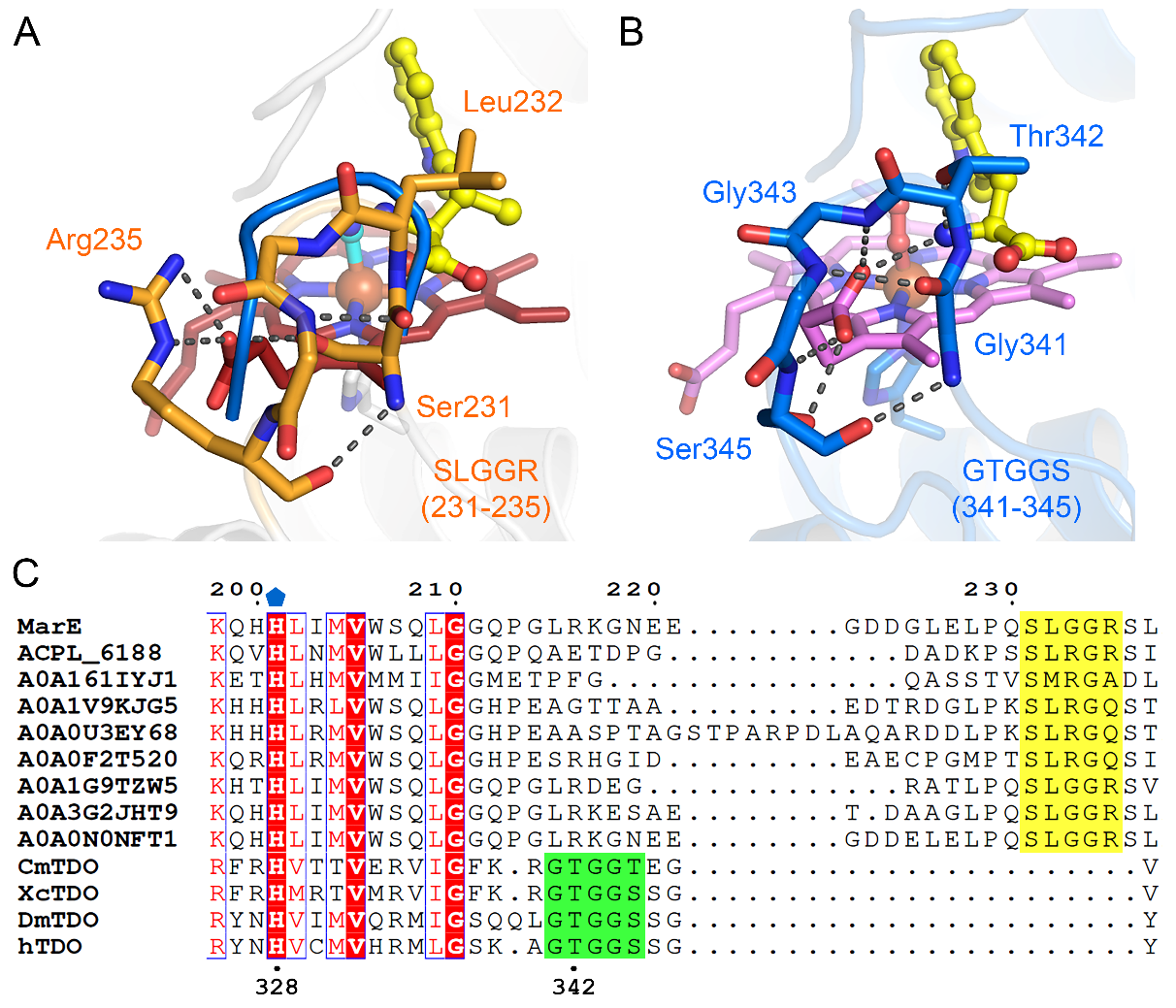


**Figure S7.** SLGGR loop of MarE and GTGGS loop of TDO. (A) SLGGR loop of MarE is shown in orange carbon color stick model with cyanide (cyan)-bound heme (dark red) and β-Me-L-Trp (yellow) (9CA3.pdb). GTGGS loop of TDO (blue) is overlaid with SLGGR loop. (B) GTGGS loop of TDO is shown in stick model (blue) with O_2_ (red)-bound heme (pink) and L-Trp (yellow) (5TI9.pdb). (C) Segment of multiple sequence alignments of MarE and TDO from various origins. Numbering at the top corresponds to MarE, while numbering at the bottom pertains to TDO. A blue solid pentagon denotes the axial heme ligand. Green boxes indicate the GTGGS loop in TDOs, and yellow boxes highlight the corresponding SLGGR in MarE.


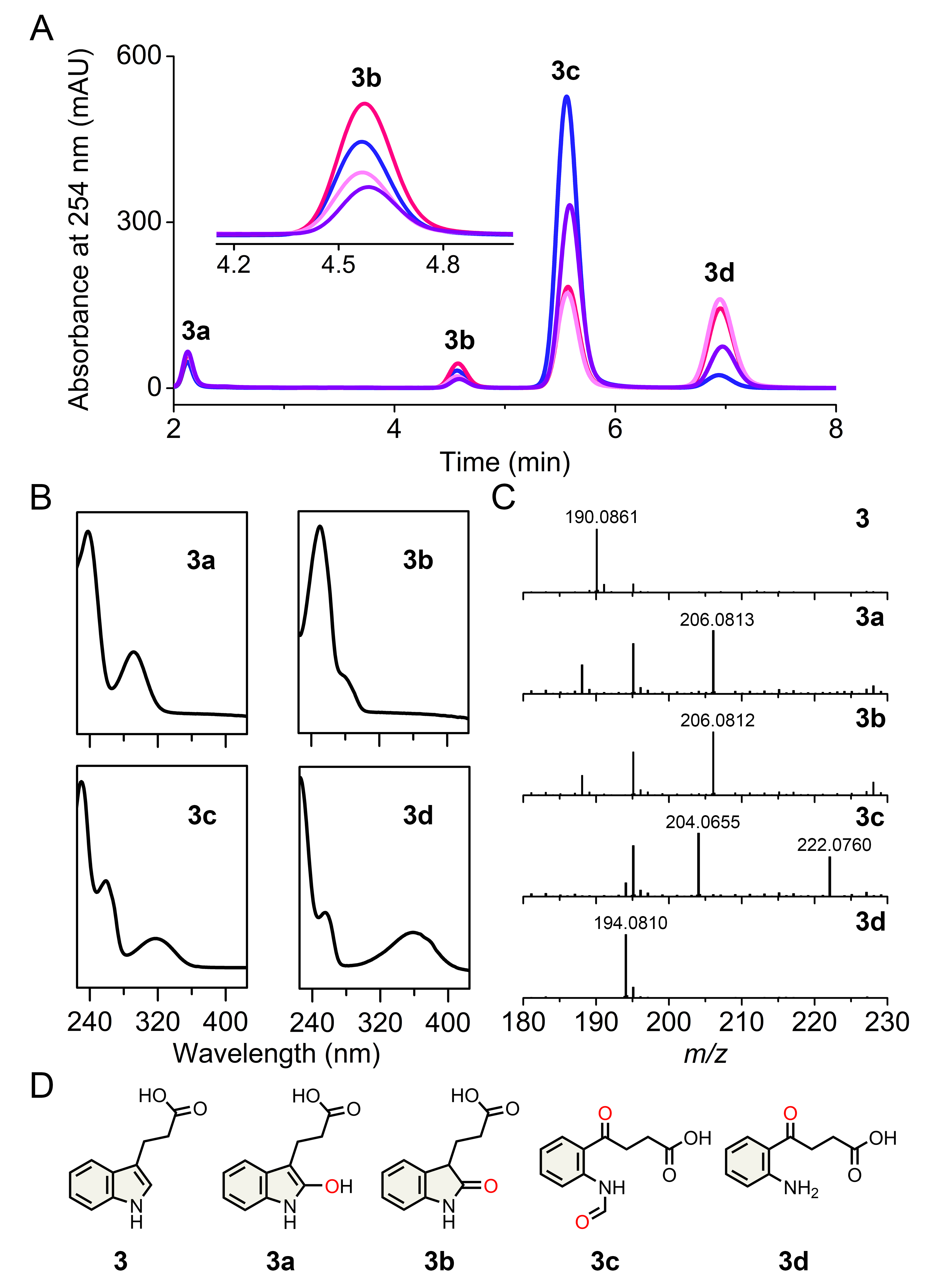


**Figure S8**. HPLC analysis for the reactions catalyzed by human TDO and variants on IPA (**3**). Chromatograms are shown for wild-type TDO (), GTGGS-to-GTGGA TDO (), GTGGS-to-GPPGS TDO (), and GTGGS-to-G_GGS TDO (). The inset is the zoomed-in view for peak **3b**. Each reaction was carried out using enzyme (50 μM), IPA (1 mM), and ascorbate (20 mM). (B) UV-vis spectra of peaks **3a**, **3b**, **3c**, and **3d**. (C) HRMS analysis of LC fractions. Calculated *m/z* values with HRMS errors in ppm are as follows: **3** (C_11_H_11_NO_2_), 190.0863 (1.05 ppm); **3a** and **3b** (C_11_H_11_NO_3_), 206.0812 (0.48 and 0 ppm); **3c** (C_11_H_11_NO_4_), 222.0761 (0.45 ppm); **3d** (C_10_H_11_NO_3_), 194.0812 (1.03 ppm). The UV-vis spectrum of **3d** has a peak centered at 358 nm and *m/z* value of 194.0810, consistent with a loss of a formyl group. (D) Chemical structures of IPA (**3**) and its oxygenation products.


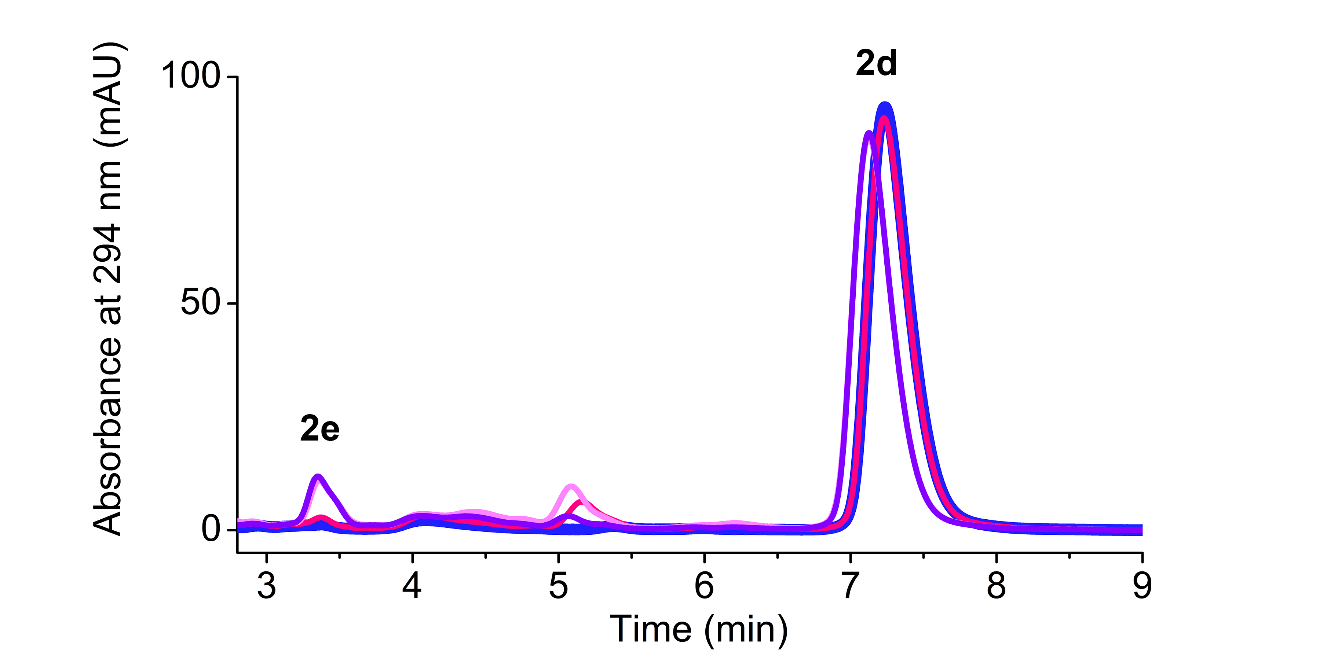


**Figure S9**. HPLC chromatograms for the reactions catalyzed by TDO and the loop variants on L-Trp (**2**). Wild-type TDO (), GTGGS-to-GTGGA TDO (), GTGGS-to-GPPGS TDO (), and GTGGS-to-G_GGS TDO (). Each reaction was carried out using enzyme (30 μM heme), L-Trp (1 mM), and ascorbate (20 mM).


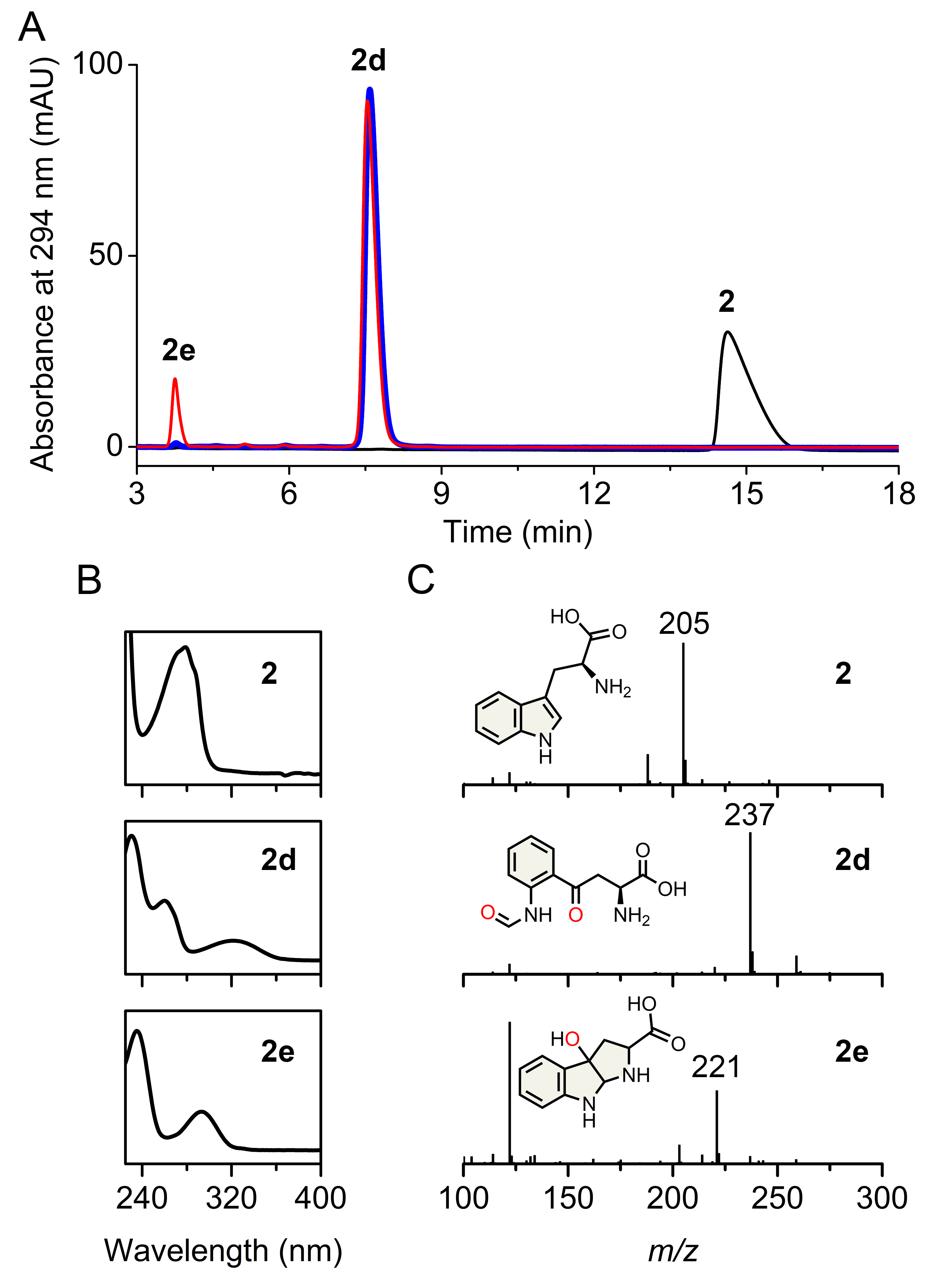


**Figure S10**. Comparisons of catalytic activities of TDO and the GTGGS-to-SLGGS variant on L-Trp (**2**). (A) The reactions catalyzed by wild-type TDO (blue trace) and GTGGS-to-SLGGS variant (red trace) with L-Trp (**2,** black trace) were analyzed by HPLC. Reactions were carried out using enzyme (50 μM heme), ascorbate (1 mM), and L-Trp (1 mM). (B) UV-vis spectra of peaks from the HPLC elution profile. (C) Mass spectra of the peaks from the HPLC elution profile and their chemical structures.


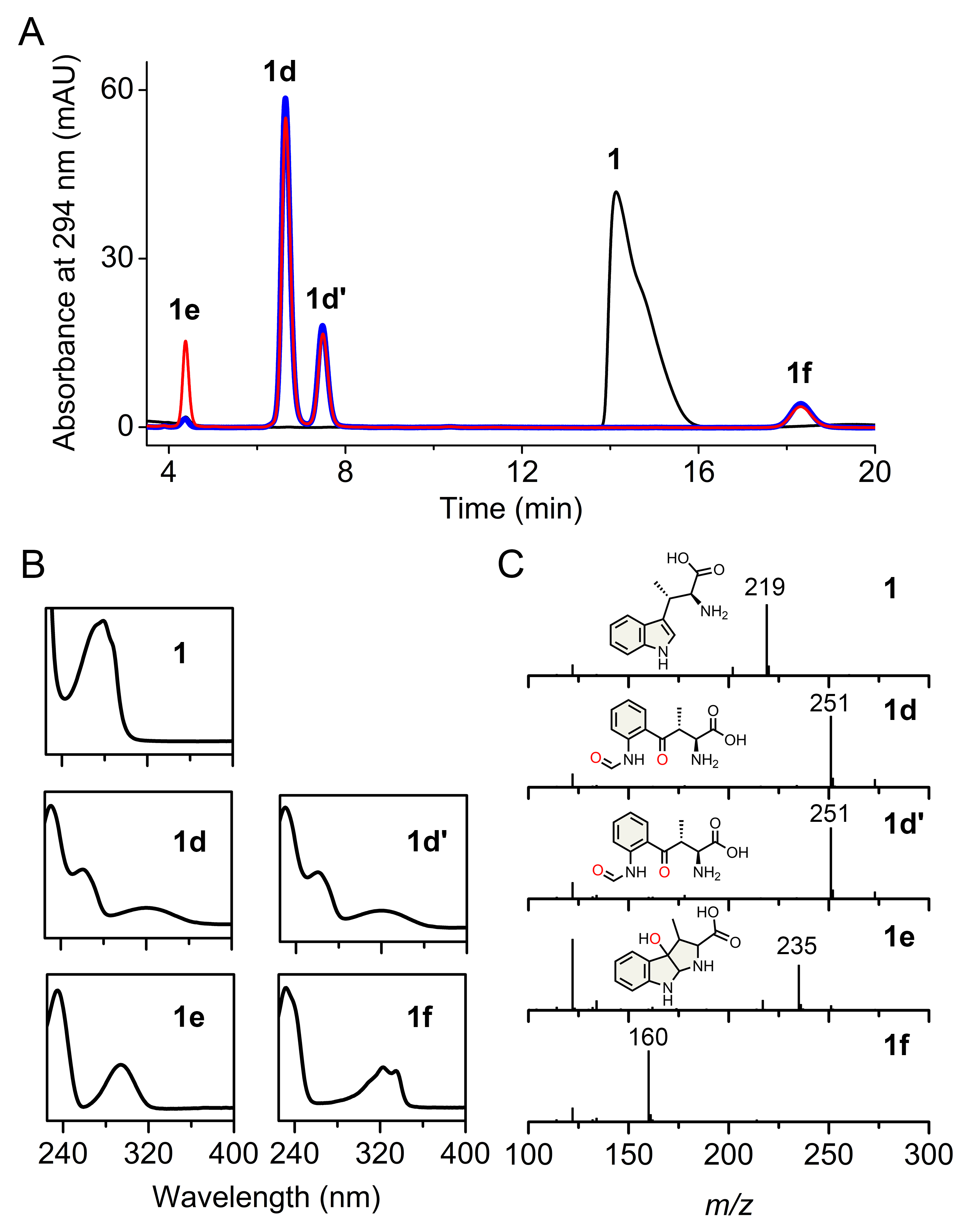


**Figure S11**. Comparisons of catalytic activities of TDO and the GTGGS-to-SLGGS TDO variant on the substrate of MarE, β-Me-L-Trp (**1**). (A) The reactions catalyzed by wild-type TDO (blue trace) and GTGGS-to-SLGGS TDO variant (red trace) with β-Me-L-Trp (**1,** black trace) were analyzed by HPLC. Reactions were carried out using enzyme (50 μM heme), ascorbate (1 mM), and β-Me-L-Trp (1 mM). (B) UV-vis spectra of the elute from the HPLC separation profile. (C) Mass spectra of the peaks from the HPLC elution profile and their chemical structures.


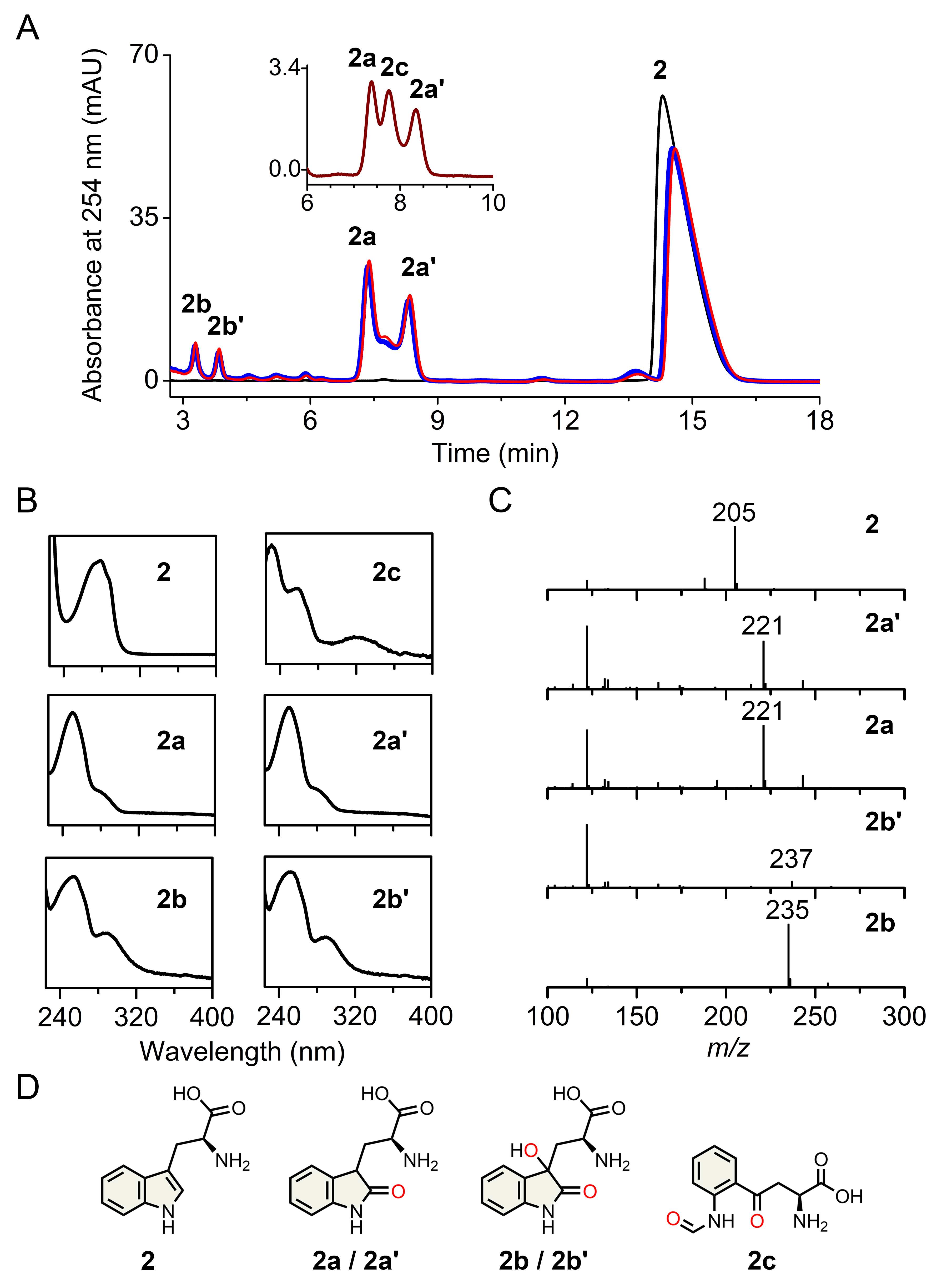


**Figure S12**. Near identical catalytic activity of MarE and the SLGGR-to-GTGGR variant on L-Trp (**2**). (A) The reactions catalyzed by MarE wild-type (blue trace) and SLGGR-to-GTGGR variant (red trace) with L-Trp (**2,** black trace) were analyzed by HPLC. Reactions were carried out using enzyme (50 μM heme), ascorbate (20 mM), and L-Trp (1 mM). Inset is the HPLC chromatogram of MarE SLGGR-to-GTGGR variant with lower concentration of ascorbate (1 mM) (dark red trace). (B) UV-vis spectra of peaks from the HPLC elution profile. (C) Mass spectra of the peaks from the HPLC elution profile. (D) Chemical structures of L-Trp (**2**) and its oxygenated products by MarE. When less ascorbate (1 mM) was used, eluant **2c** emerged between **2a** and **2aʹ** with spectral features similar to **1b** (Figure 2). The MS detection of **2c** was unsuccessful due to its significantly low intensity. Products **2b** and **2bʹ** share similar UV-vis features with an absorption maximum centered at 294 nm and a feature centered at 253 nm, which are distinct from those of **2a** and **2aʹ**. The *m/z* values of **2b** and **2bʹ** were 235 and 237.


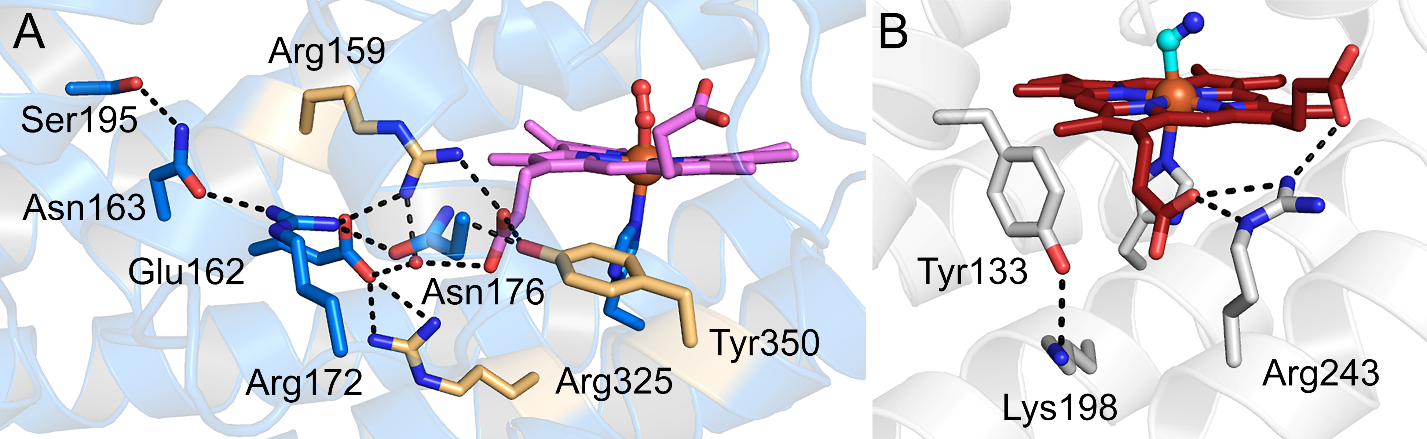


**Figure S13**. The difference in the connectivity of the down-propionate between MarE and TDO. (A) Surrounding residues near the down -propionate in TDO. Direct interactions between the down-heme-propionate and Arg159, Tyr350, and Arg325 are presented (5TI9.pdb). (B) Residues near the down-propionate in MarE (9CA3.pdb). Interactions within 3.2 Å are presented with dotted lines.


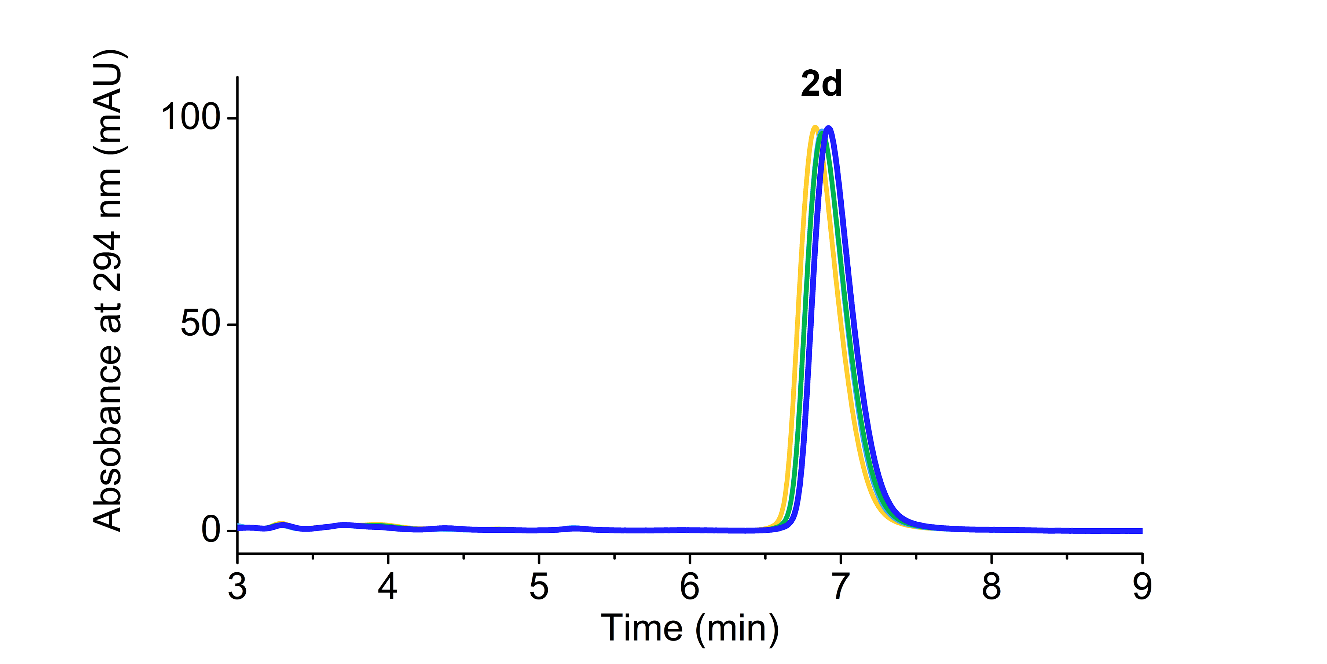


**Figure S14**. HPLC chromatograms for the reactions catalyzed by TDO variants affecting the down-propionate on L-Trp (**2**). The wild-type TDO (), R325A TDO (), R159A TDO (), and Y350F TDO (). Each reaction was carried out using the enzyme (15 μM heme), L-Trp (1 mM), and ascorbate (20 mM).


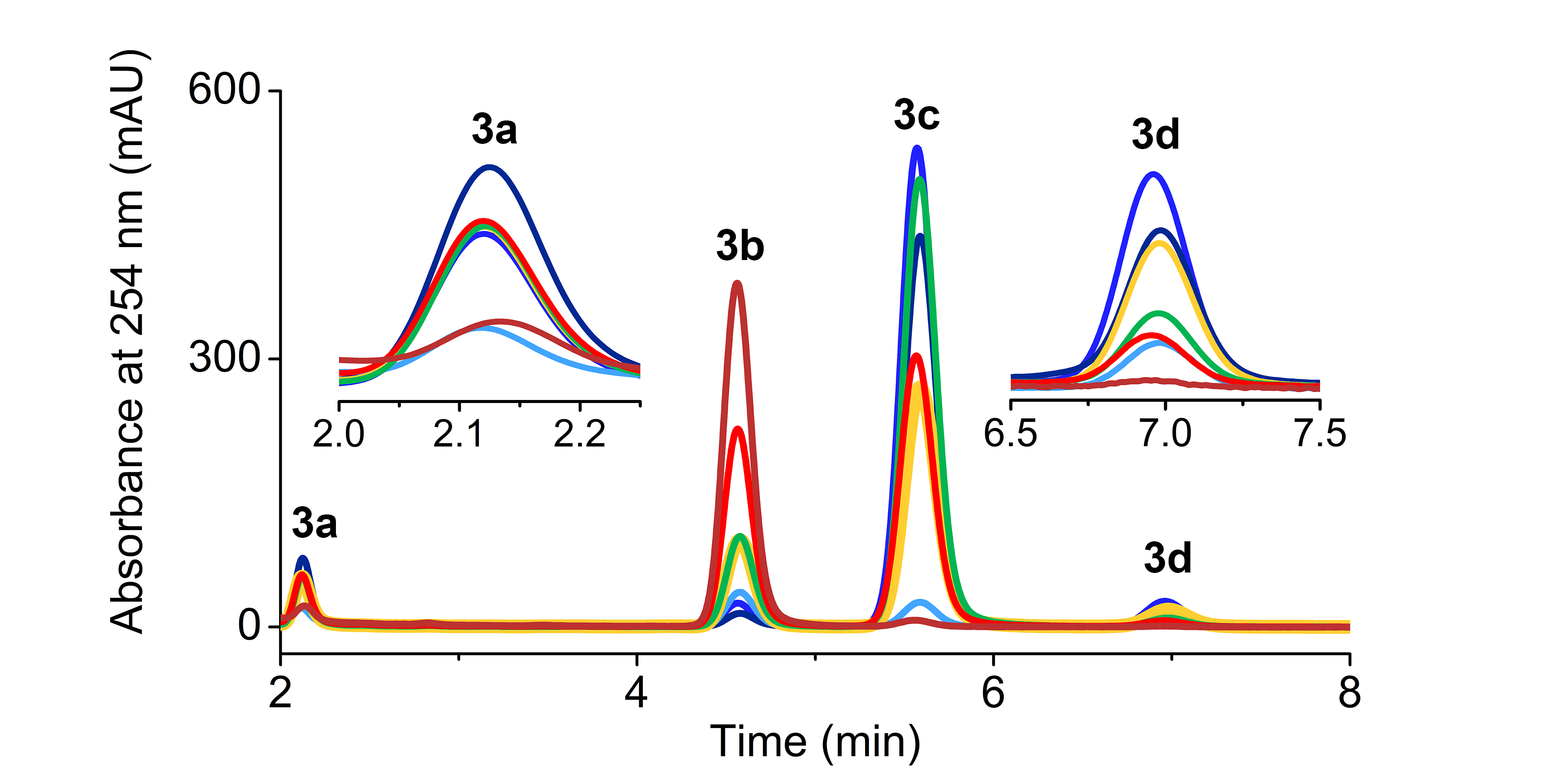


**Figure S15**. HPLC analysis for the reaction products of IPA (**3**) as an alternative substrate. (A) Chromatograms are shown for wild-type TDO (), GTGGS-to-SLGGS TDO (), R325A TDO (), R159A TDO (), Y350F TDO (), wild-type MarE (), and SLGGR-to-GTGGR MarE ().


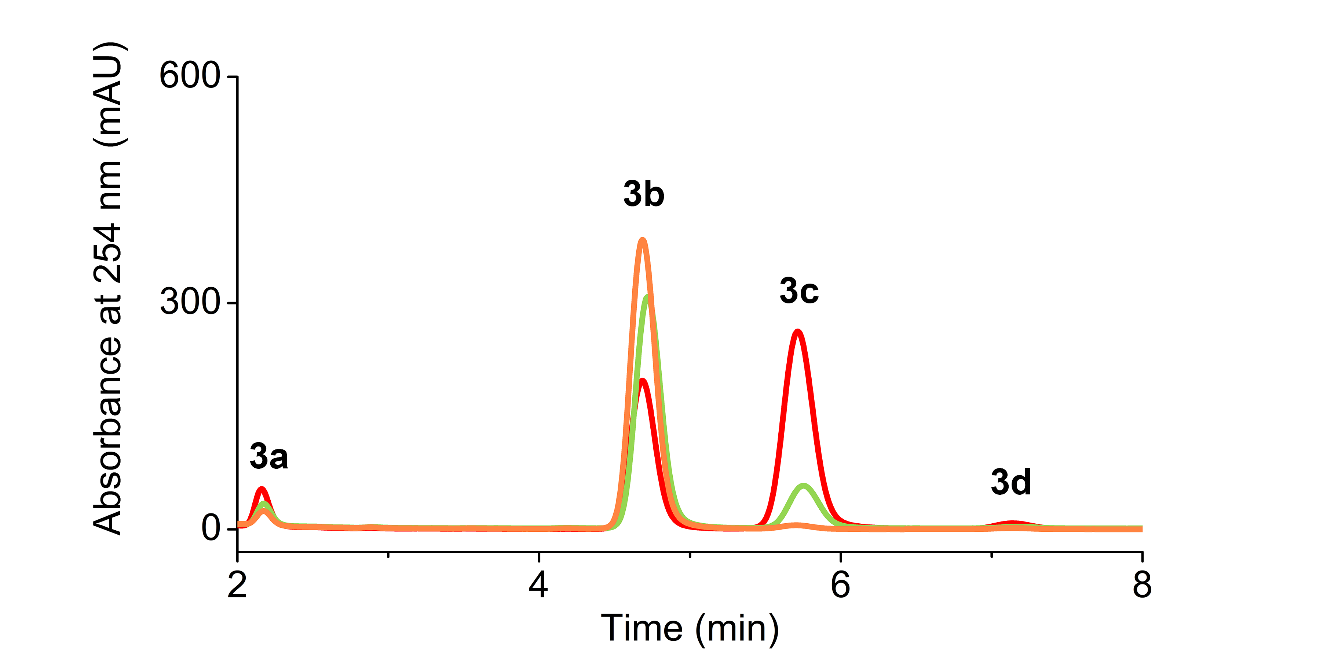


**Figure S16**. HPLC analysis for the reactions catalyzed by MarE and its loop variants on IPA (**3**). Chromatograms are shown for wild-type MarE (), SLGGR-to-SLGGS MarE (), and SLGGR-to-GTGGS MarE (). Each reaction was carried out using enzyme (50 μM), IPA (1 mM), and ascorbate (20 mM).


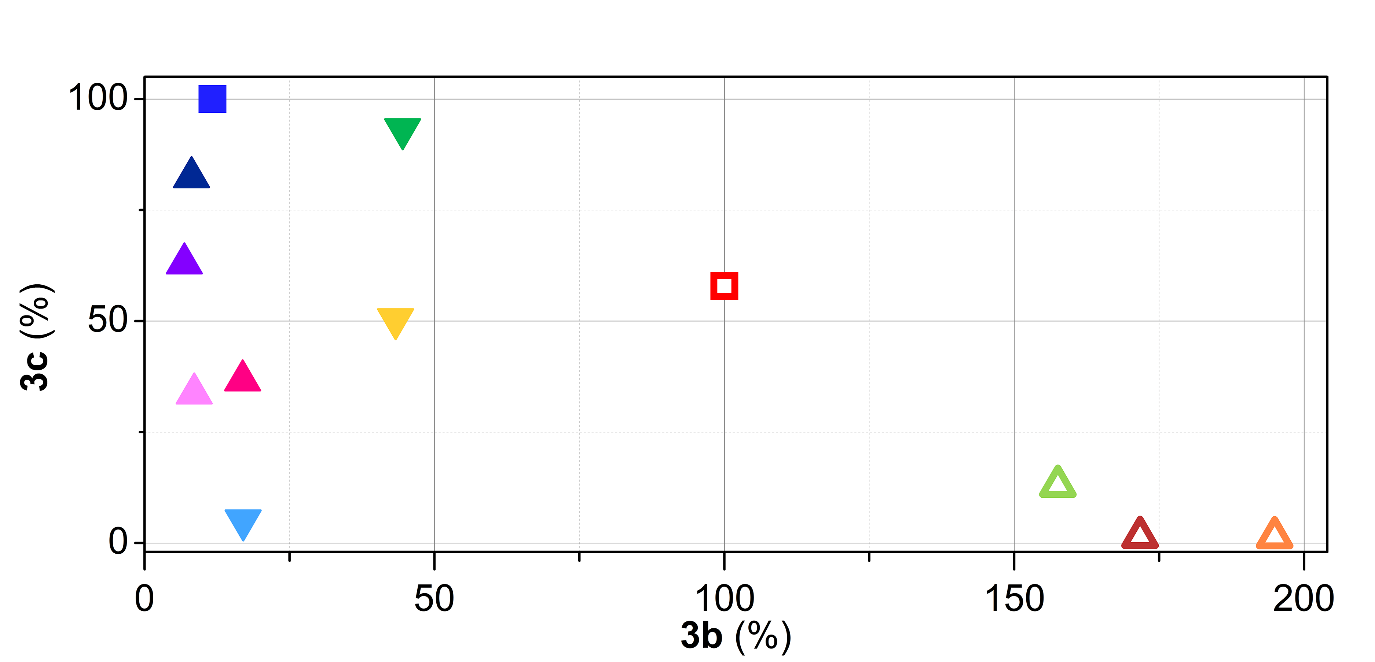


**Figure S17**. The product formation profiles between **3b** and **3c** in the reactions promoted by MarE, human TDO, and their variants. Solid and open symbols represent TDO and MarE, respectively. Square symbols stand for wild-type enzymes. Up triangles denote loop variants. Down triangles are variants for the down-propionate, which are wild-type MarE (), SLGGR-to-GTGGR MarE (), SLGGR-to-SLGGS MarE (), and SLGGR-to-GTGGS MarE (), wild-type TDO (), GTGGS-to-SLGGS TDO (), R325A TDO (), R159A TDO (), Y350F TDO (), GTGGS-to-GTGGA TDO (), GTGGS-to-GPPGS TDO (), and GTGGS-to-G_GGS TDO ().

**References cited**

[1] M. Herger, P. van Roye, D. K. Romney, S. Brinkmann-Chen, A. R. Buller, F. H. Arnold, *J. Am. Chem. Soc.* **2016**, *138*, 8388-8391.

[2] I. Barr, F. Guo, *Bio Protoc.* **2015**, *5*, e1594.

[3] I. Shin, R. C. Nguyen, S. R. Montoya, A. Liu, *J. Biol. Chem.* **2025**, *301*, 108241.

[4] Y. Zhang, Y. Zou, N. L. Brock, T. Huang, Y. Lan, X. Wang, Z. Deng, Y. Tang, S. Lin, *J. Am. Chem. Soc.* **2017**, *139*, 11887-11894.
